# Genome Diversity and Evolution in the Budding Yeasts (Saccharomycotina)

**DOI:** 10.1534/genetics.116.199216

**Published:** 2017-06-06

**Authors:** Bernard A. Dujon, Edward J. Louis

**Affiliations:** * Department Genomes and Genetics, Institut Pasteur, Centre National de la Recherche Scientifique UMR3525, 75724-CEDEX15 Paris, France; † Université Pierre et Marie Curie UFR927, 75005 Paris, France; ‡ Centre for Genetic Architecture of Complex Traits, University of Leicester, LE1 7RH, United Kingdom; § Department of Genetics, University of Leicester, LE1 7RH, United Kingdom

**Keywords:** diversity, evolution, genomes, population, yeasts, YeastBook

## Abstract

Considerable progress in our understanding of yeast genomes and their evolution has been made over the last decade with the sequencing, analysis, and comparisons of numerous species, strains, or isolates of diverse origins. The role played by yeasts in natural environments as well as in artificial manufactures, combined with the importance of some species as model experimental systems sustained this effort. At the same time, their enormous evolutionary diversity (there are yeast species in every subphylum of Dikarya) sparked curiosity but necessitated further efforts to obtain appropriate reference genomes. Today, yeast genomes have been very informative about basic mechanisms of evolution, speciation, hybridization, domestication, as well as about the molecular machineries underlying them. They are also irreplaceable to investigate in detail the complex relationship between genotypes and phenotypes with both theoretical and practical implications. This review examines these questions at two distinct levels offered by the broad evolutionary range of yeasts: inside the best-studied *Saccharomyces* species complex, and across the entire and diversified subphylum of Saccharomycotina. While obviously revealing evolutionary histories at different scales, data converge to a remarkably coherent picture in which one can estimate the relative importance of intrinsic genome dynamics, including gene birth and loss, *vs.* horizontal genetic accidents in the making of populations. The facility with which novel yeast genomes can now be studied, combined with the already numerous available reference genomes, offer privileged perspectives to further examine these fundamental biological questions using yeasts both as eukaryotic models and as fungi of practical importance.

IT has now been 20 years since the genome of the S288C laboratory strain of *Saccharomyces cerevisiae* was sequenced (Goffeau *et al.* 1996, 1997). At this time, it was the first and only eukaryotic genome entirely sequenced, and probably only few of the many participants to this scientific milestone had a clear idea of the considerable developments that would follow and their impact on almost every field of biological sciences and their applications. Today, full genome sequences are available for less than a 10th of all described yeast species (>1500 in total, see Kurtzman *et al.* 2011) and, for several of them, sequences of multiple isolates are available for comparisons. These figures are rapidly increasing, and create a novel situation in which our knowledge about basic biological mechanisms, deduced from favored experimental models such as *S. cerevisae* and *Schizosaccharomyces pombe*, can be usefully complemented with observations of the natural diversity of a group of organisms with an extremely long evolutionary history.

It is now clear that yeasts do not represent a monophyletic group of primitive, unicellular eukaryotes but have repeatedly emerged during evolution from distinct lineages of Ascomycota or Basidiomycota containing more complex forms of fungi (Nagy *et al.* 2014). If budding yeasts, the Saccharomycotina subphylum of Ascomycota to which *S. cerevisiae* belongs, represent the most successful monophyletic group of yeasts by the total number of species described (almost two thirds of all known yeasts), other yeast species are distributed between the three subphyla of Basidiomycota (representing altogether about a third of all known yeasts) and the Taphrinomycotina subphylum of Ascomycota (∼3% of total) (Kurtzman *et al.* 2011). A few unicellular or dimorphic fungi in which the unicellular form (yeast) is restricted to specific environmental conditions also exist in the Pezizomycotina subphylum of Ascomycota, otherwise comprised of filamentous fungi, as exemplified by the black yeast *Hortaea werneckii* (Lenassi *et al.* 2013) and the temperature-dependent *Talaromyces marneffei* (Woo *et al.* 2011). This unequal taxonomic distribution is further biased in presently available genomic sequences (reviewed in Dujon 2015a). If numerous data are available for the Saccharomycotina genomes, much more fragmented information exists for the other lineages despite their broader evolutionary diversity. For this reason, and also because the *YeastBook* series is devoted to *Saccharomyces*, the present review will focus on the budding yeasts, with only occasional mention of the other lineages, as appropriate for discussion. Information can be found on the genomes of other yeasts in recent publications such as Rhind *et al.* (2011) and Farrer *et al.* (2015).

The review is made of two parts. The first one focuses on *Saccharomyces*, the best-studied genus of yeasts containing, in addition to well-characterized laboratory strains, wild isolates of several species spread on all continents as well as domesticated strains and numerous hybrids. The second part explores the entire subphylum of Saccharomycotina at the genome level, trying to identify the major events that shaped its diversity across a long evolutionary range. Lessons learned at both scales remarkably converge, providing a coherent picture of the major mechanisms underlying the dynamics of eukaryotic genomes in evolving populations.

## What Did We Learn from Comparative and Population Genomics of the Saccharomyces Species Complex?

### The monophyletic *Saccharomyces* (formerly *S. sensu stricto*) clade

**Figure 1:**
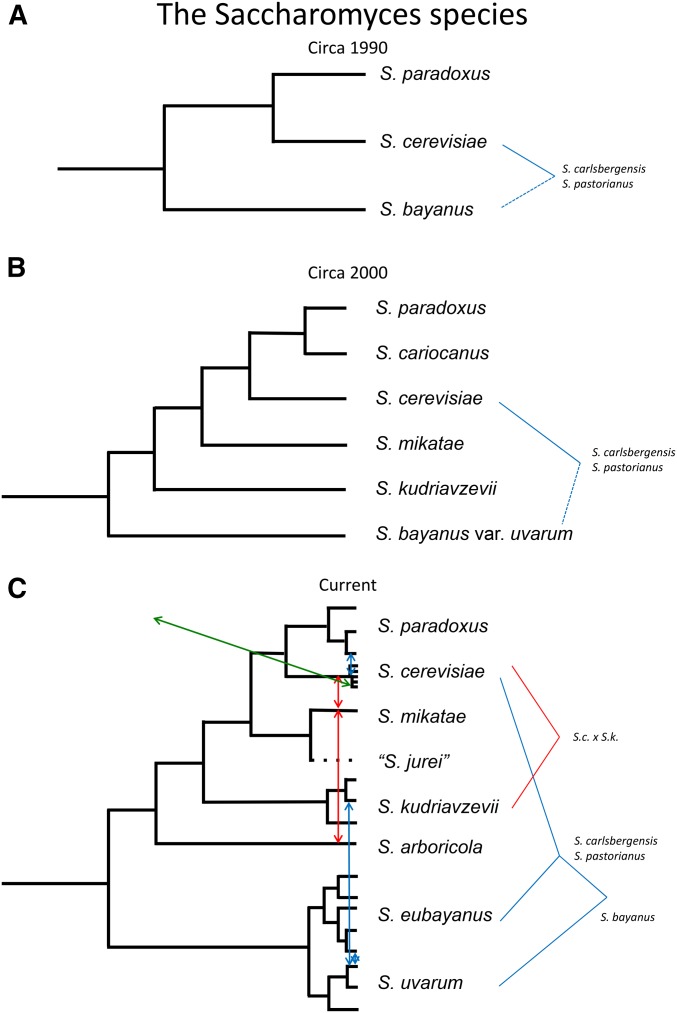
Evolution of the phylogeny of the *Saccharomyces* (formerly *S. sensu stricto*) group since the first genome sequence. (A) By the mid-1980s to mid-1990s, the use of DNA–DNA reassociation (Vaughan Martini and Martini 1987; Vaughan Martini 1989) and the biological species definition (Naumov 1987) led to the consolidation of the *Saccharomyces* yeasts into three species and one hybrid used in lager fermentation. This hybrid was between *S. cerevisiae* and something close to *S. bayanus*, but not *S. bayanus* itself (Casey and Pedersen 1988; Hansen and Kielland-Brandt 1994; Hansen *et al.* 1994). (B) By the late 1990s, the use of the biological species definition, along with electrophoretic karyotyping and presence/absence of specific repeated sequences, on isolates in various culture collections resulted in the discovery of three new species, *S. cariocanus*, *S. mikatae*, and *S. kudriavzevii*, and the refinement of *S. bayanus* var. *uvarum* as a species while *S. bayanus* itself appears to be a hybrid. (C) In recent years, whole genome sequencing along with genetic analysis has resulted in the current view of the group. One species (*S. cariocanus*) has disappeared based on phylogeny (see text) and three others have been discovered (*S. arboricola*; *S. eubayanus*, the other parent in the lager hybrids; and *S. jurei*). There are many examples of HGT, red arrows, as well as introgressions, blue arrows (see text). Perhaps the most interesting is the HGT of genes that provide useful traits in wine fermentation, green arrow, which distinguishes the wine yeast from wild European yeasts (see text and Fig. 2).

Over the decades, *Saccharomyces* species were defined by various physiological criteria, the biological species definition, and by DNA–DNA reassociation. Species appeared and disappeared in the literature until the time of the start of the yeast genome-sequencing project. Both the biological species definition (Naumov 1987) and DNA–DNA reassociation studies (Vaughan Martini and Martini 1987) resulted in the community settling on three species and the hybrid used in lager production (*S. cerevisiae*, *S. paradoxus*, *S. bayanus*, and *S. pastorianus/carlsbergensis*) (Figure 1A). The isolation and identification of new strains and perhaps new species was hampered by the lack of knowledge of the ecology and natural history of *Saccharomyces* yeasts. A survey of culture collections where budding yeasts had been isolated from various sources, using the biological species definition (interspecies sterility and intraspecies fertility), along with some molecular analysis [pulsed-field gels and ribosomal DNA (rDNA) sequences], did result in the identification of new species and hybrids such that by the turn of the millennium we had doubled their number (Figure 1B). Three new species with apparently limited ranges were described: *S. mikatae* and *S. kudriavzevii* from the Far East, and *S. cariocanus* from Brazil (Naumov *et al.* 1995a,b, 2000). *S. bayanus* appeared to contain both hybrids and a *bona fide* species called *S. bayanus* var. *uvarum* (Nguyen *et al.* 2011). Other hybrids were found, in particular a hybrid between *S. cerevisiae* and *S. kudriavzevii* used in some European wine production (Gonzalez *et al.* 2006; Lopandic *et al.* 2007).

Of the original species, *S. cerevisiae* was found in many niches, mostly associated with fermentation activities, while *S. paradoxus* was found mostly associated with oak trees around the world. *S. bayanus* was mostly found in fermentation activities as was the hybrid *S. pastorianus*. The new species were found in natural environments, associated with oak trees or insects, and had much more limited (or so was thought at the time of their discovery) geographic distributions. As most of the species could be isolated in the Far East, it was speculated that the *Saccharomyces* clade may have originated there (Naumov 1987). This has been supported by more recent surveys of diversity, where most genetic diversity for several of the species is found in the Far East as well (Wang *et al.* 2012). The genomic analysis of hybrids presented a conundrum to yeast researchers. *S. pastorianus* is a hybrid between *S. cerevisiae* and another species close to but not *S. uvarum* (Casey and Pedersen 1988; Hansen and Kielland-Brandt 1994; Hansen *et al.* 1994). The European *S. cerevisiae* × *S. kudriavzevii* hybrids presented another conundrum, namely that no European population of *S. kudriavzevii* was known, bringing into question where the hybridization took place (Hittinger *et al.* 2004). Improved sampling techniques (Sniegowski *et al.* 2002) and sampling at different temperatures (Sampaio and Goncalves 2008) along with a wider effort in environmental sampling has led to the identification both of the sister species to *S. uvarum*, called *S. eubayanus* (Libkind *et al.* 2011), and of a European population called *S. kudriavzevii* (Sampaio and Goncalves 2008). *S. eubayanus* was first identified in Patagonia which led to a further conundrum as to how the lager hybrid was formed, given that it was likely to be in use prior to Columbus’ arrival in the Americas. Further sampling across the globe has revealed populations of *S. eubayanus* in China, Tibet (Bing *et al.* 2014), New Zealand (Gayevskiy and Goddard 2016), as well as in North America (Peris *et al.* 2014), providing a way out of this historical problem. In addition to these new species and populations, other species have been discovered. *S. arboricola* was recently found in China (Wang and Bai 2008) and subsequently in New Zealand (Gayevskiy and Goddard 2016), and very recently a novel species has been found in France, provisionally called “*S. jurei*” [MycoBank registration number MB819910 (http://www.mycobank.org); Naseeb *et al.* 2017], which is a sister to *S. mikatae*. We now have three more species and have lost one (see species discussion below on *S. cariocanus* and the concepts of biological species *vs.* phylogenetic species), and have several distinct populations of many of the species. Our current understanding of the clade is shown in Figure 1C and consists of *S. cerevisiae*, *S. paradoxus*, *S. mikatae*, *S. jurei*, *S. kudriavzevii*, *S. arboricola*, *S. uvarum*, and S. *eubayanus*, along with numerous hybrids involving many of the species.

As sampling improves and sequencing becomes less expensive, we have increased our knowledge of *S. cerevisiae* and its relatives and have revealed evolutionary processes underlying the evolution of this clade. More species are being found and new populations of species previously thought to have limited geographic distributions are being discovered. This may not supplant the Far East as the origin of the clade but does lead to the conclusion that perhaps we can find *Saccharomyces* yeasts everywhere if we look hard enough. The new field of environmental DNA (Martin and Rygiewicz 2005; Thomsen and Willerslev 2015) may shed light on the true distribution of *Saccharomyces* yeasts.

### Chromosome evolution within the clade

As sequencing was still expensive and time consuming at the time of the first genome being completed, comparative analysis was done via physical- and limited-sequence comparisons. This more limited approach was still valuable in terms of determining phylogenetic relationships and evolutionary processes. Individual representatives of most of the species known at the time were sequenced [*S. paradoxus*, *S. mikatae*, *S. kudriavzevii*, and *S. uvarum* (Cliften *et al.* 2003; Kellis *et al.* 2003)], which allowed a more detailed comparison of genomes. Since then there has been a more complete, high quality assembly of these genomes (Scannell *et al.* 2011). The *Saccharomyces* clade shares a great deal of gene content and synteny, having settled down to a similar chromosome karyotype after more rapid chromosomal rearrangements and deletions following the whole-genome duplication (WGD) (Scannell *et al.* 2006). Other post-WGD species, including *Candida glabrata*, *Naumovozyma castellii*, and *Vanderwaltozyma polyspora*, have very different karyotypes having diverged from the *Saccharomyces* clade soon after the WGD (Scannell *et al.* 2006, 2007a).

Early electrophoretic karyotype analysis of some of the species (Naumov *et al.* 1992) was followed by a more thorough physical analysis of the six known species in 2000 (Fischer *et al.* 2000). Using probes designed for each chromosome arm and each centromere region, their gross chromosomal structure and breakpoints of large rearrangements between species was described. This analysis was able to map the breakpoints of several translocations relative to *S. cerevisiae* in the clade. Most of these were coincident with Ty elements or Ty LTRs. Two of the species were collinear with *S. cerevisiae* and these were not correlated with the phylogenetic relationships. This indicated that the *S. cerevisiae* chromosome configuration was likely the ancestral configuration. These rearrangements were therefore not the driver of reproductive isolation leading to speciation across the whole clade. The translocations that were mapped were nonrandomly distributed with respect to phylogenetic branch lengths and indeed appeared to have occurred in bursts, perhaps due to Ty activity. In particular, the *S. cariocanus* karyotype had four reciprocal translocations relative to the very closely related *S. paradoxus*, which is collinear with *S. cerevisiae*, the next closest relative. The idea of bursts of Ty activity has been supported by more recent population genomic sequencing (Liti *et al.* 2009a) where the Ty content of the two *S. cariocanus* isolates, with four reciprocal translocations mapped, have the highest number of Ty elements, second only to S288C; whereas most *S. paradoxus* isolates, to which *S. cariocanus* phylogenetically belongs, have lower numbers of Ty elements than the majority of *S. cerevisiae* strains. Other examples of large numbers of gross chromosomal rearrangements (GCRs), such as the Malaysian *S. cerevisiae* lineage (Liti *et al.* 2009a; Cubillos *et al.* 2011), are also likely to be due to bursts of Ty activity.

### Gene losses and differentiation of function

The individual genome sequences from one strain each of some of the species supported these earlier physical studies but also led to some interesting findings. For example, the entire galactose-utilization (GAL) pathway in *S. kudriavzevii* (Hittinger *et al.* 2004) was inactivated due to various mutations in the genes, which could have been due to the independent accumulation in each gene, leading to pseudogenes. This was found in an isolate of the species from the Far East. The more recent finding of a European population supports the nature of this loss as this population has a functioning GAL pathway (Hittinger *et al.* 2010). Finding this population also solved one of the mysteries of where yeasts originated, given that many European wine strains are *S. cerevisiae* × *S. kudriavzevii* hybrids but no European *S. kudriavzevii* population was known before. Another example where mutation accumulation between species occurs came from the use of genome annotations as a tool for comparative analysis. When the *S. uvarum* gene annotations were compared to those of *S. cerevisiae*, as many as 35 apparent breakpoints were discovered (Fischer *et al.* 2001), yet the physical analysis of the chromosomes described only 8 due to four reciprocal translocations (Fischer *et al.* 2000). This was rectified by direct sequence comparisons where relics of genes were found between open reading frames (ORFs) in one species compared to the other, resulting in loss of nearest neighbor synteny contacts when only annotated ORFs are used (Lafontaine *et al.* 2004). There can clearly be rapid accumulation in genes and sets of genes in one lineage compared to another.

As these species are derived from a WGD ancestor, there are many examples of duplicated genes, and these can lead to differentiation of function when one or the other is not lost. Two examples are the *GAL1-GAL3* (Hittinger and Carroll 2007) and the *SIR2-HST1* (Rusche and Rine 2001) pairs of duplicates. Other examples have been found in comparison of the reference genomes for each species in the clade (Scannell *et al.* 2011). Most of the differentiation occurred prior to the expansion of the clade but there is still further differentiation among the species (Kuang *et al.* 2016).

### Population genomics

The sequencing of representatives of some of these species was followed after a while by the sequencing of multiple individuals within species, and this has recently been followed by whole-genome sequencing of hundreds to thousands of individuals, mostly of *S. cerevisiae*. In the days prior to large-scale, second-generation sequencing, sample sequencing of several genes was used to look at diversity and phylogenetic relationships. A few individuals were completely sequenced for specific purposes. EC1118, a wine strain (Novo *et al.* 2009); YJM789, a clinical isolate (Wei *et al.* 2007); W303, another laboratory strain (Ralser *et al.* 2012); Sigma1278b, another laboratory strain (Dowell *et al.* 2010); and CEN.PK, an industrial strain (Nijkamp *et al.* 2012); were sequenced. This was in addition to several studies of partial sequences in *S. cerevisiae* (Fay and Benavides 2005) and *S. paradoxus* (Tsai *et al.* 2008), as well as across the clade (Liti *et al.* 2006). Whole-genome diversity was also assessed using microarrays based on the S288C sequence (Gresham *et al.* 2006; Schacherer *et al.* 2007a, 2009).

The early population genomics survey of *S. cerevisiae* led to the conclusion that there have been two domestication events, one in wine strains and one in sake strains (Fay and Benavides 2005; Schacherer *et al.* 2009). A comparison of *S. cerevisiae* variation to that seen in other species revealed much less genetic variation among *S. cerevisiae* isolates from diverse sources, which was a paradox given the phenotypic diversity that was observed. Populations of *S. paradoxus* were much more diverse from each other and correlated with geographic boundaries, with no such correlation seen in *S. cerevisiae* (Liti *et al.* 2006). The two *S. cariocanus* isolates were very close in sequence to the North American *S. paradoxus* isolates, bringing into question their designation as a species as there must have been very recent gene flow between them (Liti *et al.* 2006). Within *S. paradoxus* there was determination of the population genetics parameters of the species with a measure of how much sex and outbreeding occurred within a population (Tsai *et al.* 2008). It is clear that most evolution in the clade is by clonal expansion from vegetative reproduction with a minor contribution of sexual reproduction, however there is some sexual reproduction and recombination and they can have a great deal of influence.

The first large-scale sampling of genomes from *S. cerevisiae* as well as *S. paradoxus* was done using Sanger sequencing and resulted in the start of increased efforts into population genomics surveys (Liti *et al.* 2009a). At the same time, a microarray survey was done on a larger number of *S. cerevisiae* strains (Schacherer *et al.* 2009). The two studies were consistent with each other and there was some overlap in the strains analyzed. The main findings included: there are several distinct populations of *S. cerevisiae* that are equidistant from each other across their genomes, so called clean lineages; there are several mosaic strains that appear to be the result of recent outbreeding between these clean lineages; and that genome wide there is nothing special about the wine or sake populations, even if they have specific variants associated with adaptation to the fermentation process in which they are usually found (Perez-Ortin *et al.* 2002). One hypothesis is that the fermentation properties desired already existed in extant populations of yeast and that our activity sampled and selected the strains with the best properties. The mosaic strains clearly had some sequence variation that came from outside the five clean populations described, indicating that there were likely more populations to be discovered. The survey was consistent with the previous sample sequencing and found that the *S. paradoxus* populations were clean with little or no gene flow between them. The originally characterized *S. cariocanus* was clearly embedded within the North American population based on shared sequence variation, despite the reproductive isolation.

### Clean lineages *vs.* mosaics

The concept of clean lineages merits some additional discussion, especially in *S. cerevisiae*. Clean lineages were defined as those populations where the phylogenetic relationship with other populations was the same across the genomes, where the topology of the phylogenetic tree remains the same for any segment of the genome being compared (Liti *et al.* 2009a). This would be expected for populations that are evolving independently with little or no gene flow between them, as is seen for the *S. paradoxus* populations in which the genetic diversity is completely correlated with geographic location. The survey of 36 *S. cerevisiae* genomes revealed that about half fell into five clean lineages by this definition, while the other half were comprised of segments from two or more of the clean lineages with a few segments from unknown populations. As many of these mosaic strains were associated with human activity—such as bread making, food spoilage, fermentation, and even clinical cases—the origins of these recent outbred strains could be due to human activity providing the opportunity for interbreeding. The contrast with *S. paradoxus* could be a partial explanation for the higher levels of phenotypic diversity seen in *S. cerevisiae* despite lower overall genetic diversity. The genetic diversity that exists in *S. cerevisiae* is more mixed up due to outbreeding, which may result in an expansion of the phenotypic space.

Such a global genome analysis has not yet been done on other populations, so it is not known whether the wild populations from China (Wang *et al.* 2012) or those associated with other human activities (Cromie *et al.* 2013; Arana-Sanchez *et al.* 2015; Ludlow *et al.* 2016) are clean lineages or mosaics, though admixture was detected using genome-wide analysis of variants using restriction site associated DNA sequencing (Cromie *et al.* 2013; Ludlow *et al.* 2016). It would be very interesting if some populations associated with human activity are mosaic with genetic combinations that make them useful for particular activities, such as bread making. It is clear that some fermentation-associated populations are clean lineages, such as the sake and the wine populations. A large number of *S. cerevisiae* strains are now sequenced or about to be sequenced (Strope *et al.* 2015b; http://1002genomes.u-strasbg.fr/index.html; http://www.ncyc.co.uk/yeast-treasure-trove-goes-live/) and this collection will provide an unparalleled view of diversity within the species, addressing issues of selection and movement by human activity as well as origins of particular adaptations.

### Genetic *vs.* phenotypic diversity

Another result of the survey was confirmation that there was greater genetic diversity in *S. paradoxus* than in *S. cerevisiae*. This result was surprising as the strains were all tested for growth under a large number of conditions and it was found that the phenotypic diversity of *S. cerevisiae* was much greater than that of *S. paradoxus*. Despite this paradox, there was a significant correlation between genetic diversity and phenotypic diversity in the two species. One hypothesis explaining this paradox is the level of outbreeding seen in *S. cerevisiae*. Perhaps the outcrossing, likely facilitated by human activity, has led to the increased phenotypic diversity by bringing together novel combinations of variants at different loci.

**Figure 2:**
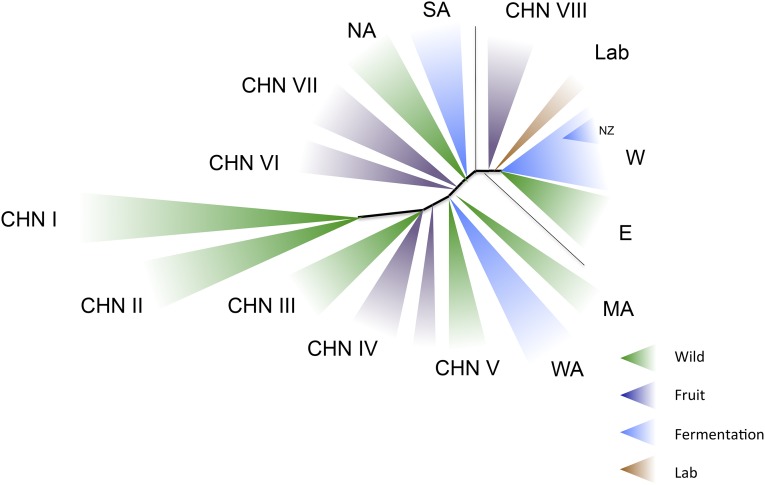
The populations of *S. cerevisiae* as an unrooted cladogram (not to scale) composite from several studies (Liti *et al.* 2009a; Wang *et al.* 2012; Almeida *et al.* 2015). Populations shown include North American (NA), sake (SA), West African (WA), Malaysian (MA), and wine/European (W) as previously defined (Liti *et al.* 2009a), with E as the wild European sister clade to the wine group, recently described (Almeida *et al.* 2015). Eight additional populations from China (CHN I–CHN VIII) were recently described (Wang *et al.* 2012). In addition a New Zealand (NZ) subpopulation of the wine population has been described (Gayevskiy and Goddard 2012). The original distinction of two domestication events leading to wine and sake fermentation (Fay and Benavides, 2005; Cromie *et al.* 2013; Arana-Sanchez *et al.* 2015; Ludlow *et al.* 2016) has been extended as more populations have been sampled. These include wild populations from oak and related trees, populations associated with fruit trees, and populations associated with fermentation activities. It is clear that many of the populations associated with fermentation are very close to wild populations exemplified by the wine *vs.* European sister groups, which differ (Almeida *et al.* 2015) in the presence/absence of the genes acquired by HGT from outside the clade (Novo *et al.* 2009). The wine population has been spread around the world from its European origins and in some cases, such as New Zealand (Gayevskiy and Goddard 2012), the subpopulation can be placed within the larger population. There are now descriptions of populations associated with ale fermentation not distinguished here (Gallone *et al.* 2016; Goncalves *et al.* 2016). Lab, laboratory.

More diversity has been found in *S. cerevisiae* with the discovery of at least 10 new populations from China, many from primeval forests (Wang *et al.* 2012). Whole-genome analysis has yet to be completed but it is clear there is greater diversity in these isolates as seen in Figure 2. Some of the populations from China are close to those previously described (Liti *et al.* 2009a), but others, particularly the ones from primeval forests (populations CHN I, II, and III in Figure 2) nearly doubles the known diversity. In addition, distinct populations of *S. cerevisiae* are being found, which are associated with other fermentation activities such as cacao (Cromie *et al.* 2013; Arana-Sanchez *et al.* 2015; Ludlow *et al.* 2016) and coffee (Ludlow *et al.* 2016) processing.

There are now thousands of *S. cerevisiae* isolates from a variety of sources. Many cluster with their source even if not with location. Figure 2 is a composite of *S. cerevisiae* populations sampled over the years and analyzed by different means. The most sampled population is the wine/European, which has subpopulations found around the world wherever wine is produced. One subpopulation analyzed extensively is that from New Zealand which must have arrived with human migration (Goddard *et al.* 2010; Gayevskiy and Goddard 2012). A sister population was recently found; it is associated with oaks in Europe but not used in fermentation (Almeida *et al.* 2015). This strain differs by the lack of alien DNA from other species that confers useful properties for wine making (Novo *et al.* 2009). There are numerous wild populations from around the world as well as distinct populations associated with various human fermentation activities including beer, chocolate, coffee, *etc*. (Legras *et al.* 2007; Sicard and Legras 2011; Tapsoba *et al.* 2015; Ludlow *et al.* 2016). There are also intermediate populations associated with fruit trees and orchards. As can be seen in Figure 2, almost every population associated with fermentation or fruit trees has a close wild sister population.

As more populations of different species are being found, whole-genome surveys are being expanded, and now there is good data on populations of *S. uvarum* (Almeida *et al.* 2014), *S. eubayanus* (Almeida *et al.* 2014; Peris *et al.* 2014, 2016a), and *S. kudriavzevii* (Hittinger *et al.* 2010; Peris *et al.* 2016b), in addition to *S. cerevisiae* and *S. paradoxus*. These will make for interesting comparisons with *S. cerevisiae*, particularly for those species with little human influence.

### Domestication or harnessing existing potential

Given the large number of surveys of *S. cerevisiae* from a wide variety of locations and environments, we can address the issue of domestication more thoroughly. The wine/European population is the most widespread due to human wine production, and the introduction and spread of these yeasts can be observed in some cases (Gayevskiy and Goddard 2012, 2016). The question of domestication, at least with the wine/European population, can be informed by the very recent discovery of a wild population from oak trees that is sister to the wine/European population (Almeida *et al.* 2015). This finding supports the hypothesis that rather than domestication, our use in fermentation took advantage of the properties of an existing population of yeast with perhaps some adaptations to the wine-making process (see below for discussion of introgressions). However, there are phenotypes and specific genetic variants associated with use in wine fermentation (Gallone *et al.* 2016). In ale and lager strains, there is stronger evidence of selection on specific genes (Gallone *et al.* 2016; Goncalves *et al.* 2016) as well as on the whole genome (Baker *et al.* 2015) from sequence comparisons. Of the other species used in human fermentation activities, there are probably not enough populations sampled to address the domestication issue. Many strains used in fermentation are hybrids of other *Saccharomyces* species with *S. cerevisiae* (Figure 1C), and it may be that the existence of hybrids preceded their use in fermentation.

### Subtelomeres and the pan-genome

One thing that became apparent from whole-genome sequence surveys is that you cannot study what you do not know exists. With microarrays and other hybridization techniques, you can only assess variation in known sequences. Several genes and gene families were known from yeast studies that are not found in the S288C genome. Six of these gene families involved in carbon-source utilization are located in the subtelomeres (Liti *et al.* 2009a). Subtelomere variation and dynamics was already recognized (Louis and Haber 1990, 1992; Louis *et al.* 1994; Louis 1995) and it was known that the region was a hotbed of gene-family evolution (Brown *et al.* 2010). The analysis of the *Saccharomyces* Genome Resequencing Project collection revealed 38 new gene families not previously known to be in *S. cerevisiae*, in addition to the six known families (Liti *et al.* 2009a). These varied in presence/absence as well as in copy number across the *S. cerevisiae* and *S. paradoxus* isolates. It is clear that as a species, *S. cerevisiae* is much more than merely represented by S288C.

This pan-genome has become important in the study of genetic variation underlying phenotypic variation among strains. A recent set of tools has been developed to help assess genetic material not found in the reference genome (Song *et al.* 2015). Quantitative genetic studies have found that much of the causal genetic variation for a given trait maps beyond the ends of assembled contigs into the subtelomeres. In any given strain, there may be ∼8% of the genome that is considered subtelomeric. In most quantitative traits analyzed, up to 25% of the causal segregating variation maps to the subtelomeres (Cubillos *et al.* 2011; Liti and Louis 2012). One explanation of the higher phenotypic diversity on *S. cerevisiae* compared to *S. paradoxus* is subtelomeric variation. A total of 50% of the phenotypic variation difference can be explained by copy number and presence/absence variation in subtelomeric genes, which is greater in *S. cerevisiae* than in *S. paradoxus* (Bergstrom *et al.* 2014). This leaves 50% to be explained perhaps by outbreeding, supported by the expansion of the phenotypic variation in every cross between clean lineages as described above.

### Horizontal gene transfers

The sequence of a wine strain, EC1118, revealed several genome segments whose origin was from outside *S. cerevisiae*, but within the *Saccharomyces*, and some outside the clade (Novo *et al.* 2009; Marsit *et al.* 2015). These segments conferred useful properties to the wine niche and therefore may be considered adaptive. One of these appears to have originated from a wine spoilage yeast and so it is possible that somehow DNA from this was incorporated into a wine strain either by some level of cellular interaction or by uptake from the environment. It is certainly possible that the two were occupying the same vessel at the same time during fermentation. The structure of the insertions in various strains indicated a circular intermediate that integrated independently in different configurations (Borneman *et al.* 2011; Galeote *et al.* 2011). All wine strains have some permutation of this configuration and the other horizontal gene transfers (HGTs), and so it has spread through the yeast used by the wine community. Another example is in Ty2 elements, which are not thought to be infective though they do generate virus-like particles within the yeast cell. *S. cerevisiae* and *S. mikatae* have many copies, yet *S. paradoxus* in between has none. Either *S. paradoxus* has lost Ty2 or never had it. The comparison consensus sequences of Ty2 in *S. cerevisiae* and *S. mikatae* indicate a time of divergence much more recently than the rest of the genome, and more recently than *S. paradoxus* and *S. cerevisiae* diverged (Liti *et al.* 2006). The most consistent explanation is that Ty2s moved from one to the other in the recent past, postspeciation.

### Hybrids, introgressions, and reticulate evolution

One of the surprising findings of population genomics surveys is the introgression of sequence (homologous replacement) seen between species. Such introgression requires the ability to hybridize, which all members of the clade have, and some breakdown of the reproductive isolation allowing gene flow from one species into the other. The first examples were between *S. paradoxus* and *S. cerevisiae*. When one genome sequence of *S. paradoxus* was available it became clear that there was a segment with little (0.1%) divergence compared to the S288C sequence, whereas the rest of the genome was 10–15% divergent (Liti *et al.* 2006). Upon analysis of the region in several isolates, it was shown that the entire European population of *S. paradoxus* had an introgression from *S. cerevisiae* not seen in the other populations. Interestingly, this was in a subtelomere. Following this observation, introgressions in both directions have been found in surveys of *S. cerevisiae* and *S. paradoxus* (Muller and McCusker 2009). As more populations of different species are surveyed, more examples of such introgressions are found (Almeida *et al.* 2014). For example, there are numerous introgressions of *S. uvarum* into *S. eubayanus* and vice versa (Peris *et al.* 2014). There are introgressions from *S. kudriavzevii* into these two species as well. Given that these species are used in human fermentation activities, the potential opportunity for genetic exchange exists.

There are numerous examples of hybrids between yeast species (see *What Did We Learn from Comparative Genomics of Other Saccharomycotina?*). They are generally sterile and therefore dead ends in terms of long-term evolution, as no sexual recombination is possible anymore. The question then arises as to how segments from one species can move into the genome of another, clearly via homologous recombination, when the hybrids are sterile? The usual thought of how this occurs is through a rare viable spore backcrossing with one of the parents, with subsequent backcrosses becoming easier and easier. The alternative is a single step homologous introgression in a hybrid that does not have to undergo numerous backcrosses, as has been observed in artificial *S. cerevisiae* × *S. uvarum* hybrids (Dunn *et al.* 2013).

The evolution of the *Saccharomyces* clade appears to be reticulate rather than bifurcating as is usually portrayed in phylogenetic trees. Within populations there is clearly mostly vegetative reproduction. However, there is some outbreeding and sexual recombination so that within a population there is breakdown of linkage disequilibrium and some level of outcrossing (Tsai *et al.* 2008). Between populations there is less gene flow, however there is admixture in some cases and certainly the movement of yeast through human activity has provided the opportunity for gene flow between populations. This is reticulate evolution and expected for sexually reproducing species. We generally think that once speciation has occurred then gene flow stops and we move from reticulate to bifurcating phylogenies, but the population genomics surveys of *Saccharomyces* species indicates otherwise. Perhaps gene flow is possible, even if rare, across the whole of the clade, leading to connections between even distant branches. This requires the exploration of reproductive isolation and its role in speciation.

### Reproductive isolation and speciation

What is a species and how will we move into the future with species concepts? We have seen above that under one definition, the biological species definition, we define *S. cariocanus* as a new species distinct from the others in the clade. However under another definition, the phylogenetic species definition, we place these isolates into the American population of *S. paradoxus*. There are numerous examples of individuals and populations having GCRs relative to closely related other strains, which would make them a species under one definition but not the other. An example is the Malaysian population of *S. cerevisiae*. It has not been labeled as a new species but as *S. cerevisiae* based on its sequence and physiological characteristics, yet it has at least 10 breakpoints from GCRs that preclude fertility when crossed to any other *S. cerevisiae* (Liti *et al.* 2009a; Cubillos *et al.* 2011). If *S. cariocanus* is a new species then this population should also be one. If it remains *S. cerevisiae*, then *S. cariocanus* should be relabeled *S. paradoxus*.

Speciation is generally thought to arise from reproductively isolated populations that continuously diverge from each other. There are three major hypotheses for the underlying mechanism of speciation, two of which are found in other systems and are well established. As discussed above there are numerous examples of introgressions between species, indicating that reproductive isolation is not complete which may bring us to question the concept of species in these yeasts (Louis 2011).

#### GCRs

It is clear that there are many GCRs segregating within populations of a species and these can result in partial to near complete reproductive isolation, which could then lead to eventual speciation. Is this a major driver of speciation within the *Saccharomyces* clade? In other Ascomycetes clades, such as the *Lachancea* clade (Vakirlis *et al.* 2016), there are large numbers of GCRs between each pair of species and it cannot be ruled out that GCRs could have been an important driver of the speciation process. In the *Saccharomyces* clade, there are several species that are collinear yet are species by all definitions, and so GCRs could not have been involved in their speciation. There are segregating translocations and large inversions that do cause spore inviability within species and populations and it may be that they are leading to eventual speciation (Hou *et al.* 2014), but as a clade these are not the major drivers of speciation. Even in cases with many GCRs such as the South American *S. paradoxus* *vs.* the North American population, or the Malaysian population of *S. cerevisiae* *vs.* other populations, there is clear evidence of gene flow as they share SNPs with one population but not others (Liti *et al.* 2009a).

#### Bateson–Dobzhansky–Müller incompatibilities

The search for speciation genes has not been very successful in many taxa, and in the *Saccharomyces* clade there are only a few examples of Bateson–Dobzhansky–Müller (B-D-M) incompatibilities leading to inviability. Dominant B-D-M incompatibilities between species have been ruled out by testing the meiotic fertility of allotetraploids (Greig *et al.* 2002a,b) where hybrid diploids exhibit little or no spore viability, but tetraploidy restores high levels of spore viability. Individual chromosome replacements of *S. paradoxus* into *S. cerevisiae* failed to find any recessive B-D-M incompatibilities (Greig 2007, 2009). In some pairs of species, a nuclear–mitochondrial incompatibility was found (Lee *et al.* 2008; Chou and Leu 2010) but it is not clear whether this arose postspeciation or not (Louis 2009, 2011). Recently a classical B-D-M incompatibility pair was found segregating in *S. cerevisiae* (Hou *et al.* 2015). This was between a nonsense mutation in a nuclear-encoded mitochondrial gene and a transfer RNA (tRNA) suppressor of the mutation. This example would never lead to speciation as the tRNA suppressor would have many detrimental pleiotropic effects. Strong divergent selection in laboratory evolution experiments can lead to B-D-M incompatibilities, as has been demonstrated (Dettman *et al.* 2010), and so there is potential for such in nature.

Despite this paucity of inviable B-D-M incompatibilities, there are numerous examples of incompatibilities resulting in lack of function of important processes. The first example was in the mismatch-repair system where a particular combination of segregating variants of *PMS1* and *MLH1* products in *S. cerevisiae*, which act together as a heterodimer, exhibited a null phenotype (Heck *et al.* 2006). Similarly segregating variants of *YKU70* and *YKU80* in *S. paradoxus* exhibited a null phenotype in one specific combination (Liti *et al.* 2009b). In crosses between the clean lineages of *S. cerevisiae*, two of six pairwise combinations resulted in a global reduction of homologous recombination during meiosis, with one of these exhibiting reduced spore viability due to loss of genetic interference (Cubillos *et al.* 2011). In any species, such as in the *Saccharomyces* clade, where populations diverge from each other yet maintain coadaptation within, it might be expected that incompatibilities arise for combinations of variants that break up coadapted gene complexes (Liti and Louis 2012). Whether these will be drivers of reproductive isolation leading to speciation remains to be demonstrated.

#### Sequence divergence and mismatch repair

A third mechanism that applies across the entire clade is simple sequence divergence and the action of the mismatch-repair system (Liti *et al.* 2006; Louis 2011). Here the intermediates of homologous recombination; required for crossing over, chiasmata formation, and proper chromosome segregation; contain numerous sequence differences between diverged parents. These are recognized by the mismatch-repair system and rather than being “repaired” to one or the other parental sequence, as would happen for a single mismatch, the large number of differences lead to abortion of the intermediate. The end result is no recombination and aneuploidy due to random segregation of the chromosomes (Chambers *et al.* 1996; Hunter *et al.* 1996). The test of this hypothesis is the restoration of some spore viability associated with increased crossing over when the mismatch repair system is deleted (Chambers *et al.* 1996; Hunter *et al.* 1996). This notion holds for the closely related species *S. paradoxus* and *S. cerevisiae* (Chambers *et al.* 1996; Hunter *et al.* 1996) but also for diverged populations within each of the species (Greig *et al.* 2003), indicating that it is acting at the right stage for incipient speciation.

**Figure 3:**
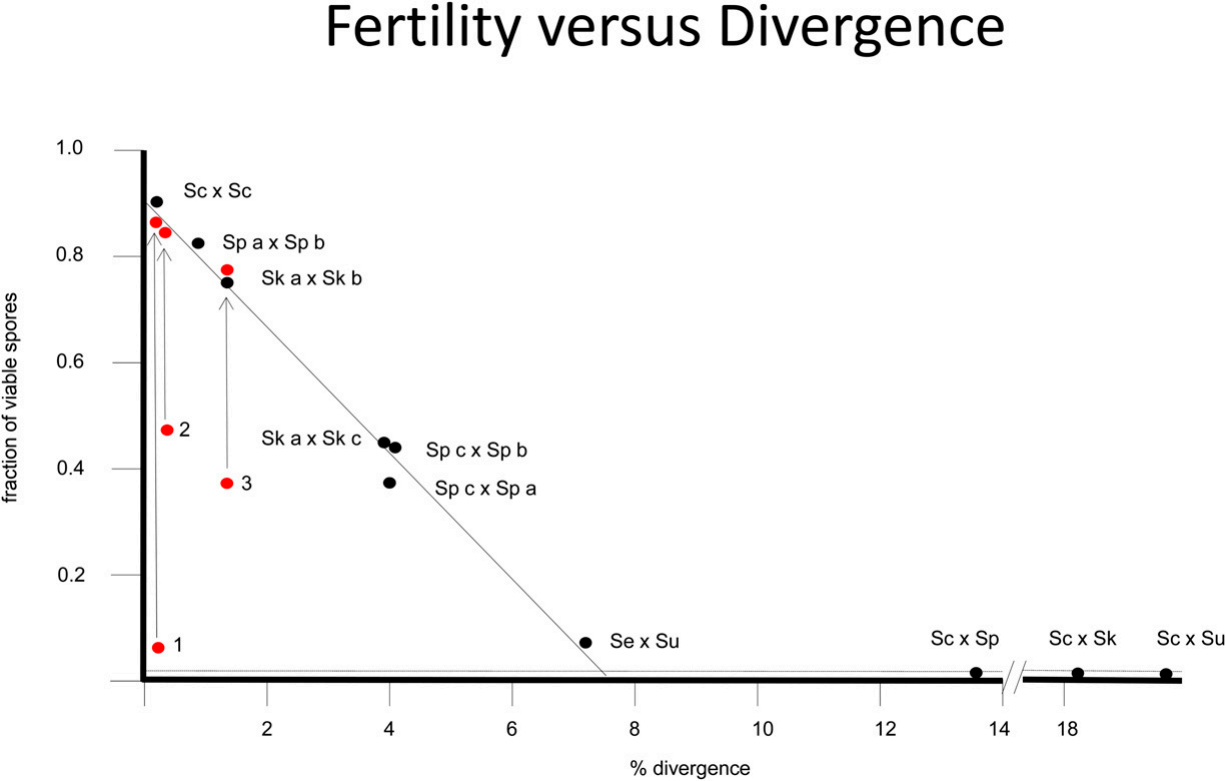
Fertility (spore viability) *vs.* sequence divergence. The biological species definition is based on within-species fertility but between-species sterility after mating. The potential causes of postzygotic reproductive isolation include GCRs, B-D-M incompatibilities, and sequence divergence itself acted upon by the mismatch-repair system during meiotic recombination. Most of the interspecies hybrids exhibit levels of spore viability that are in the range of random segregation of chromosomes leading to an accidental viable combination of <1%. In populations that have diverged yet are not separate species [three populations of *S. paradoxus* (Sp a, b, and c) and three populations of *S. kudriavzevii* (Sk a, b, and c)] as well as in the hybrid of the very closely related species *S. uvarum* and *S. eubayanus*, there is a linear relationship between sequence divergence and spore viability (Liti *et al.* 2006; Hittinger 2013). Some close strains by sequence do exhibit spore viability less than expected by the linear relationship; however, when corrected for known translocations, the viability returns to the expected relationship. This is particularly relevant for the *S. cariocanus* by North American *S. paradoxus* (1) where the sequence divergence is small and there are four previously described reciprocal translocations (Fischer *et al.* 2000) and other large GCRs discovered by complete assembly of the genome (D. Delneri, personal communication). Other examples where the correction of GCRs reestablishes the sequence divergence-spore viability relationship include strains of *S. paradoxus* from the Far East population (2) where one has a translocation, and strains from the European population and Far East populations differing by one translocation (3).

In Figure 3 there is a clear linear relationship between sequence divergence and fertility/spore viability. The off-line examples in specific crosses are accounted for by one or more reciprocal translocations and, when corrected for these, the expected fertility fits the linear relationship. In a recent survey of many isolates (Hou *et al.* 2014), where 60 were crossed to the S288C background to measure spore viability, the conclusion was that sequence divergence was not correlated with the level of spore viability and that GCRs were the major mechanism underlying the observations. There are two problems with this conclusion. One is that they surveyed a narrow range of divergence (0.1–0.5%) where not much change in viability is expected and the divergence in each measure of viability would be greater than any signal of reduced viability with increased divergence. The second and more important issue is that the S288C strain is a mosaic sharing large segments of the genome with low sequence divergence with most other isolates rather than evenly distributed divergence. These low diversity regions will allow normal recombination and chromosome segregation, alleviating any problems elsewhere in the genome.

### Remaining issues raised by *Saccharomyces*

The yeast community needs to deal with the definition of species. On the one hand, the biological species definition provides a functional definition, which coincides with phylogeny in the majority of cases. On the other hand, there are reproductively isolated populations whose isolation is due to GCRs yet there has not been sufficient time for accumulated sequence divergence. Phylogenetically, *S. cariocanus* is in the American population of *S. paradoxus* yet is reproductively isolated by infertility. Similarly, the Malaysian population of *S. cerevisiae* is reproductively isolated yet has not been designated as a new species. Perhaps the definition has to be a practical one fit for the specific purpose at hand.

Are species truly reproductively isolated? There is increasing evidence of rampant reticulate evolution in the *Saccharomyces* yeasts, with introgressions jumping across long-established separated lineages. These examples are clear evidence of the opportunities for interbreeding and indeed hybrids are found for many pairs of the species in various sets of samples. At what stage of reproductive isolation do we decide we have a species? Perhaps the level of divergence where the spore viability is as low as that of random segregation of chromosomes would be one molecular measure of phylogenetic species. Although the frequencies of such reticulate evolution in different taxa have not been analyzed sufficiently for comparison, there is evidence that such gene flow is more prevalent in many taxa than previously thought (Mallet *et al.* 2016).

What is domestication? Are the *Saccharomyces* yeasts used in fermentation activities like dogs while the wild cousins are wolves, or is the clade more like cats moving in and out of feral and tamed niches? It is clear in the wine population of *S. cerevisiae* that the utility in wine is partly due to the introgression and HGT of genes from within and outside the clade, conferring useful properties such as those beneficial to fermentation (Marsit *et al.* 2015). A wild sister population exists in Europe which is not tamed.

What is the genome of a species? It is clear that as a whole, *S. cerevisiae* has many more genes/sequences than present in the first genome sequenced. How can we describe this pan-genome and how relevant is it to understanding yeast ecology, evolution, and adaptation to specific niches, in particular those associated with human activity? How prevalent is presence/absence and copy number variation in subtelomeres of the other *Saccharomyces* species and of Ascomycetes yeasts as a whole?

## What Did We Learn from Comparative Genomics of Other Saccharomycotina?

### The rise of comparative yeast genomics: a brief history

Considerable progress in our understanding of yeast genomes and their evolution has also been made over the last decade by comparison between distantly related species. This strategy, however, faced several difficulties at the start, not the least of which being the application of relevant criteria for appropriate taxon sampling in a field traditionally dominated by practical considerations in which ecology, metabolic properties, or pathogenic characters of each yeast attracted greater attention than the evolution of their genomes. Even the definition of species in yeasts has remained an open question until now, and their taxonomy has considerably evolved over time. Today, >1500 yeast species have been described and classified in a variety of lineages (Kurtzman *et al.* 2011), of which only a subset has been studied at the genomic level so far. In addition, when the complete sequence of the *S. cerevisiae* genome was determined, back in 1996 (Goffeau *et al.* 1996), most scientists involved in this project were still deeply influenced by the concept of the universality of life that formed the basis of early molecular biology and, consequently, they were paying little attention to natural diversity and its evolutionary origin (see Dujon 2015b). What was true for *S. cerevisiae* had to also be true for other yeasts and, beyond, for other eukaryotes as well. Logically, therefore, *S. cerevisiae* immediately became the system of choice to develop functional genomics, a very successful endeavor indeed (Giaever and Nislow 2014), but it provided no insight on the evolutionary origin of its genome. For many people, it was not obvious what could be learned from the comparison of other yeasts in absence of experimental tools for many of them. Following this same logic, the second yeast genome ever completely sequenced was the other favored experimental model of yeast molecular geneticists, the fission yeast *S. pombe* (Wood *et al.* 2002), a representative of the Taphrinomycotina subphylum of Ascomycota. Beside its great intrinsic interest, this genome was, however, much too different from that of *S. cerevisiae* to identify many common traces representative of evolutionary conservation from their very distant common ancestry and to distinguish them from convergence; a phenomenon more frequent than generally anticipated (see below).

**Figure 4:**
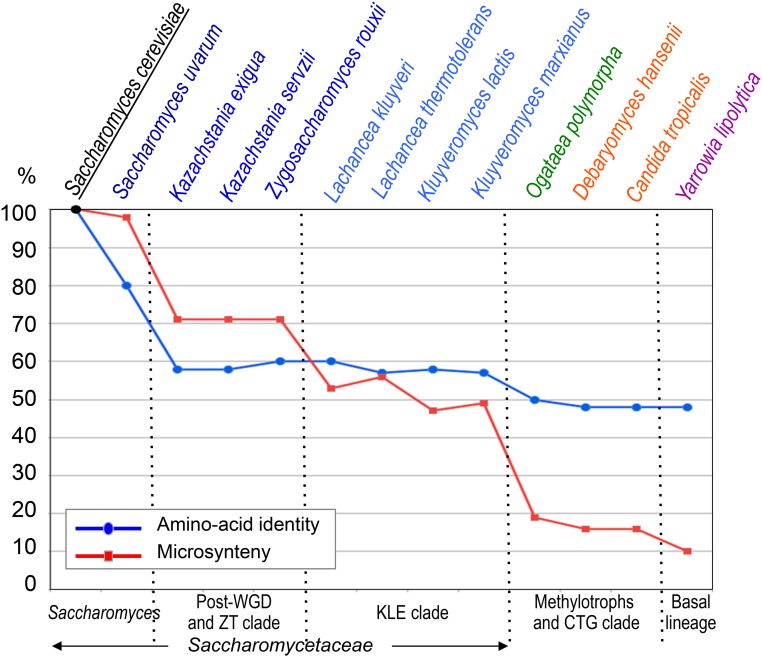
The first exploration of the evolutionary diversity of Saccharomycotina genomes, from back in 2000. Data taken from Llorente *et al.* (2000), Malpertuy *et al.* (2000), and Souciet *et al.* (2000). The figure summarizes the mean amino-acid identity (blue line) and conservation of gene adjacency (red line) when random sequence tags from each of the yeast species indicated at top were compared to *S. cerevisiae*. Ordinate in percentage. Vertical lines point to major thresholds, separating groups of species whose corresponding affiliation to present subgroups of Saccharomycotina is indicated at bottom.

In the end, it took no less than 8 years after the completion of the *S. cerevisiae* sequencing project before the first complete sequences of other Saccharomycotina genomes, seven in total, became available for analysis and comparisons. Actually, some of these species had been selected for reasons other than to examine genome evolution (see below). Yet, altogether, these genomes happened to represent interesting samples across the evolutionary range of Saccharomycotina and, consequently, often served as references in subsequent publications. This favorable situation was due in part to the results of an earlier exploration of this subphylum in which 13 different species had been sequenced at low coverage, as permitted by the technology of this time, and compared to *S. cerevisiae* (Souciet *et al.* 2000). This work gave us the first quantitative estimates of the evolutionary spectrum covered by the Saccharomycotina based on sequence divergence between orthologous genes (Malpertuy *et al.* 2000) and loss of microsynteny (Llorente *et al.* 2000). The presence of thresholds in the distributions observed (Figure 4) were the first clues about the existence of several subgroups of species worthy of further examination. If *S. uvarum* showed high sequence conservation with *S. cerevisiae* (∼80% mean amino-acid identity between orthologous gene products), the figure rapidly dropped to <60% for seven other yeasts and to <50% for the last four. Similarly, the stepwise loss of microsynteny (average gene adjacency) separated the *Kazachstania* and *Zygosaccharomyces* group (with figures close to 70% conservation) from the *Lachancea* and *Kluyveromyces* group (∼50%), and from the other yeasts (with figures dropping below 20%).

**Figure 5:**
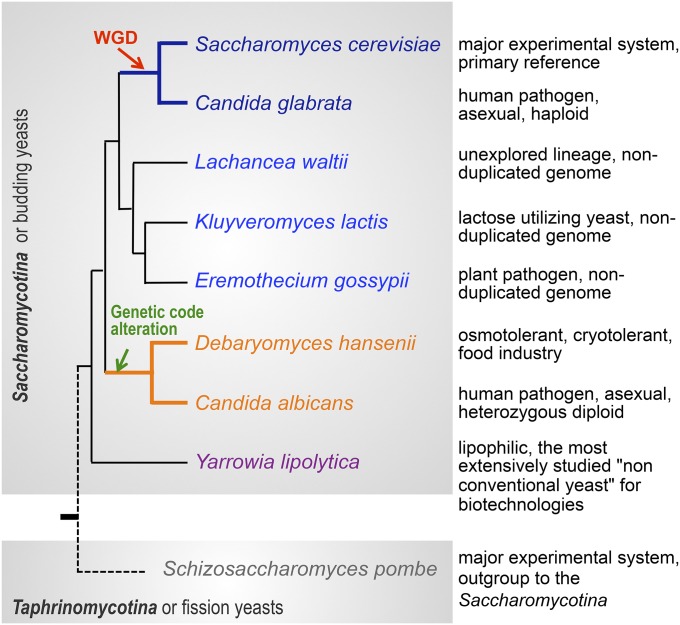
The historical set of fully sequenced yeast genomes, from back in 2004. The figure lists the nine fully sequenced yeast genomes published by year 2004 (original references in text) with indication of their major selection criterion. Tree topology from Kurtzman *et al.* (2011), branch lengths ignored.

The first eight complete genome sequences of Saccharomycotina, plus *S. pombe* used as outgroup, are summarized on Figure 5 with indication of the major criterion used for their selection: *Candida albicans* (Jones *et al.* 2004) and *C. glabrata* (Dujon *et al.* 2004) are human pathogens; *Kluyveromyces lactis*, *Debaryomyces hansenii*, *Yarrowia lipolytica* (Dujon *et al.* 2004), and *Eremothecium gossypii* (Dietrich *et al.* 2004) have biotechnological or agricultural importance. Only *Lachancea waltii* (Kellis *et al.* 2004) was primarily selected as representative of a novel lineage. Initially, *L. waltii*, *E. gossypii*, and *C. albicans* were each compared alone against *S. cerevisiae*, and the first two revealed a characteristic dual synteny supporting the idea that *S. cerevisiae* inherited from WGD followed by extensive gene loss, as initially proposed by Wolfe and Shields (1997). The last four, *C. glabrata*, *K. lactis*, *D. hansenii*, and *Y. lipolytica*, allowed the first multidimensional comparisons of yeast genomes at a global scale and revealed major signatures of the distinct lineages. Combining these data, it became clear that the human pathogen *C. glabrata*, the plant pathogen *E. gossypii*, the lactose-utilizing yeast *K. lactis*, as well as *L. waltii*, shared many genomic features with *S. cerevisiae*; features now known as characteristics of the Saccharomycetaceae family (see below). Alternatively, the human pathogen *C. albicans*, the halotolerant and occasional pathogen *D. hansenii*, and the methylotrophic, alkane-utilizing yeast *Y. lipolytica*, representative of other families, showed very distinct characteristics including, for the first two, the usage of the alternative genetic code (see Santos *et al.* 2011) and for the last one a larger and less compact genome (see Kelkar and Ochman 2011).

As the importance of genomics rapidly grew in subsequent years, additional yeast genomes were sequenced including, for the earliest ones: *Scheffersomyces stipitis*, a xylose-fermenting yeast (Jeffries *et al.* 2007); *V. polyspora*, representing an early branching lineage after the duplication (Scannell *et al.* 2007b); *Komagataella phaffii*, the commonly used host for heterologous protein production previously designated *Pichia pastoris* (De Schutter *et al.* 2009; Mattanovich *et al.* 2009); *Lachancea thermotolerans*, *Lachancea kluyveri*, and *Zygosaccharomyces rouxii*, three other Saccharomycetaceae that did not inherit the WGD (Souciet *et al.* 2009); and several pathogenic *Candida* (*C. albicans*, *C. tropicalis*, *C. dubliniensis*, and *C. parapsilosis*) plus the related species *Lodderomyces elongisporus*, *Meyerozyma guillermondi*, and *Clavispora lusitaniae* (Butler *et al.* 2009; Jackson *et al.* 2009). Other, incomplete genome sequences were also released during the same period, some revealing interspecies hybridizations. By 2010, >20 Saccharomycotina species had been fully sequenced (Dujon 2010). This number has now risen to >100 (ignoring the many hybrids) if one only counts the completely assembled sequences and permanent drafts with sufficiently limited numbers of scaffolds (Table 1, Table 2, and Table 3). Many more are lying in the form of published draft sequences with too large numbers of scaffolds to be useful for genome analyses, or remain publicly nonavailable. A project to sequence the genomes of all described yeast species is planned (Hittinger *et al.* 2015).

**Table 1:** Major reference genomes of the Saccharomycetaceae and related families

Species	Strain	Genome size (Mb)*^a^*	No. of chromosomes*^b^*	Total no. of CDS*^c^*	Reference or source
**Saccharomycetaceae (post-WGD)**					
*** Saccharomyces cerevisiae***	S288C	**12.2**	**16**	5771	Goffeau *et al.* (1996)
*** Saccharomyces paradoxus***	CBS432^T^	**12.0**	**16**	5527	unpublished data*^d^*
* Saccharomyces mikatae*	IFO1815^T^	(11.4)	**16**	6384	Scannell *et al.* (2011)
*** Saccharomyces kudriavzevii***	IFO1812^T^	(11.3)	**16**	5968	Scannell *et al.* (2011)
* Saccharomyces arboricola*	CBS10644^T^	(11.6)	**16**	5413	Liti *et al.* (2013)
*** Saccharomyces uvarum***	CBS7001	(11.5)	**16**	5915	Scannell *et al.* (2011)
*** Saccharomyces eubayanus***	CBS12357^T^	(11.7)	**16**	5515	Baker *et al.* (2015)
*** Candida glabrata***	CBS138^T^	**12.3**	**13**	5203	Dujon *et al.* (2004)
* Candida bracarensis*	CBS10154^T^	(12.2)	**12**	5315	Gabaldón *et al.* (2013)
* Nakaseomyces delphensis*	CBS2170^T^	(10.7)	**10**	5168	Gabaldón *et al.* (2013)
* Candida nivariensis*	CBS9983^T^	(11.5)	**13**	5238	Gabaldón *et al.* (2013)
* Candida castelii*	CBS4332^T^	(10.1)	**8**	4875	Gabaldón *et al.* (2013)
* Nakaseomyces bacillisporus*	CBS7720^T^	(10.9)	**15**	5086	Gabaldón *et al.* (2013)
* Kazachstania africana**^e^*	CBS 2517^T^	**11.1**	**12**	5378	Gordon *et al.* (2011)
* Kazachstania naganishii**^e^*	CBS8797^T^	**10.8**	**13**	5321	Gordon *et al.* (2011)
* Naumovozyma castellii**^e^*	CBS 4309^T^	**11.2**	**10**	5592	Gordon *et al.* (2011)
* Naumovozyma dairenensis**^e^*	CBS 421^T^	**13.5**	**11**	5548	Gordon *et al.* (2011)
* Tetrapisispora phaffii**^e^*	CBS 4417^T^	**12.1**	**16**	5250	Gordon *et al.* (2011)
* Tetrapisispora blattae**^e^*	CBS 6284^T^	**14.0**	**10**	5389	Gordon *et al.* (2011)
* Vanderwaltozyma polyspora**^e^*	DSMZ70294^T^	(14.7)	(41)	5652	Scannell *et al.* (2007a,b)
**Saccharomycetaceae (ZT clade)**					
*** Zygosaccharomyces rouxii***	CBS732^T^	**9.8**	**7**	4997	Souciet *et al.* (2009)
*** Zygosaccharomyces bailii***	CLIB213^T^	(10.3)	(27)	5084	Galeote *et al.* (2013)
*** Torulaspora delbrueckii****^e^*	CBS1146^T^	**9.2**	**8**	4972	Gordon *et al.* (2011)
**Saccharomycetaceae** (**KLE clade**)					
* Lachancea fantastica* nom. nud.	CBS6924	**11.3**	**7**	5060	Vakirlis *et al.* (2016)
* Lachancea meyersii*	CBS8951^T^	**11.3**	**8**	4997	Vakirlis *et al.* (2016)
* Lachancea dasiensis*	CBS10888	**10.7**	**8**	5099	Vakirlis *et al.* (2016)
* Lachancea nothofagi*	CBS11611^T^	**11.3**	**8**	5153	Vakirlis *et al.* (2016)
*** Lachancea thermotolerans***	CBS6340^T^	**10.4**	**8**	5177	Souciet *et al.* (2009)
* Lachancea quebecensis*	CBS14088	(10.2)	(51)	5075	Freel *et al.* (2016)
*** Lachancea waltii***	NCYC2644	(10.2)	(8)	(4768)	Kellis *et al.* (2004)
* Lachancea lanzarotensis*	CBS12615^T^	(11.5)	(24)	5058	Sarilar *et al.* (2015)
* Lachancea mirantina*	CBS11717	**10.1**	**8**	5057	Vakirlis *et al.* (2016)
* Lachancea fermentati*	CBS6772	**10.3**	**8**	5233	Vakirlis *et al.* (2016)
* Lachancea cidri*	CBS2950	**10.1**	**8**	5188	Vakirlis *et al.* (2016)
*** Lachancea kluyveri***	CBS3082^T^	**11.3**	**8**	5378	Souciet *et al.* (2009)
* Kluyveromyces lactis*	CLIB210	**10.6**	**6**	5108	Dujon *et al.* (2004)
* Kluyveromyces dobzhanskii*	CBS 2104	(10.7)	(86)	4957	B. Nystedt and S. Astrom; unpublished data*^f^*
*** Kluyveromyces marxianus***	NBRC1777	**10.9**	**8**	4912	Inokuma *et al.* (2015)
—	DMKU3-1042	**11.0**	**8**	4952	Lertwattanasakul *et al.* (2015)
*** Eremothecium gossypii***	ATCC10895	**8.7**	**7**	4718	Dietrich *et al.* (2004), (2013)
* Eremothecium cymbalariae*	DBVPG 7215	**9.7**	**8**	4712	Wendland and Walther. (2011)
* Eremothecium coryli*	CBS5749	(9.1)	**6**	4682	Wendland and Walther, (2014)
* Eremothecium aceri*	FM-2008	**8.9**	**7**	4479	Dietrich (2013)
* Eremothecium sinecaudum*	ATCC58844	**8.9**	**7**	4528	F. S. Dietrich; unpublished data*^g^*
**Saccharomycodaceae**					
* Hanseniaspora opuntiae*	AWRI3578	(8.8)	(18)	4176	Sternes *et al.* (2016)
* Hanseniaspora osmophila*	AWRI3579	(11.4)	(17)	4660	Sternes *et al.* (2016)
* Hanseniaspora uvarum*	AWRI3580	(8.8)	(18)	4061	Sternes *et al.* (2016)
**Wickerhamomycetaceae**					
* Wickerhamomyces anomalus*	NRRL Y-366-8	(14.1)	(46)	6423	Riley *et al.* (2016)
*** Cyberlindnera jadinii***	NRRL Y-1542	(13.0)	(76)	6038	Riley *et al.* (2016)
* Cyberlindnera fabianii*	JCM3601	(12.3)	(12)	5874	R. Manabe - unpublished data*^h^*
**Saccharomycopsidaceae**					
* Saccharomycopsis malanga*	JCM7620	(16.7)	(44)	6280	R. Manabe, R. Endoh, S. Uzuhashi, G. Okada, M. Takashima and M. Ohkuma; unpublished data*^h^*
* Saccharomycopsis fibuligera*	KPH12		**7**	6155	Choo *et al.* (2016)

The table lists the yeast species of indicated families whose genomes were published in the form of complete sequences or permanent drafts with reasonable numbers of scaffolds (<100). Exploratory sequences (low coverage), sequence read archives, and preliminary assemblies with excessive numbers of contigs or scaffolds were ignored. Readers can find some of these additional sequences with their references in Hittinger *et al.* (2015). Strain numbers correspond to the reference indicated. Species in which several isolates have been sequenced in complete form or permanent drafts are indicated by bold type, and only one isolate is listed (the first one or the best one). Note that an exception was made for *K. marxianus* because two independent sequences of equivalent quality were published simultaneously. Species designation follows Kurtzman *et al.* (2011) and may, therefore, differ from original publications. Hybrid genomes and unspecified isolates are ignored.

a Genome sizes are indicated in bold type when determined from complete sequences and in brackets when deduced from scaffolds in assemblies (figures are haploid equivalent in case of known diploid strains). Sizes ignore rDNA, mtDNA, and plasmids.

b Numbers of chromosomes are indicated in bold type when known. Figures in brackets correspond to numbers of scaffolds in assemblies.

c Total numbers of predicted protein-coding genes (CDS) are taken from original publications or subsequent annotations, as most appropriate. Figures in brackets represent estimate from incomplete sequence.

d Unpublished data taken from https://yjx1217.github.io/Yeast_PacBio_2016/data/.

e Sequence data taken from http://ygob.ucd.ie.

f Unpublished data taken from GenBank: CCBQ000000000.1.

g Unpublished data taken from GenBank: CP014248.1.

h Unpublished data taken from the National BioRecource Project (www.jcm.riken.jp/cgi-bin/nbrp/nbrp_list.cgi).

**Table 2:** Major reference genomes of the CTG clade (Debaryomycetaceae, Metchnikowiaceae, and related species)

Species	Strain	Genome size (Mb)*^a^*	No. of chromosomes*^b^*	Total no. of CDS*^c^*	Reference or source
**Debaryomycetaceae**					
*** Debaryomyces hansenii***	CBS767^T^	**12.2**	**7**	6411	Dujon *et al.* (2004)
* Priceomyces haplophilus*	JCM1635	(10.5)	(9)	5253	R. Manabe, R. Endoh, S. Uzuhashi, G. Okada, M. Takashima and M. Ohkuma unpublished data*^d^*
* Meyerozyma guilliermondii*	ATCC6260	(10.6)	(8)	6135	Butler *et al.* (2009)
* Meyerozyma caribbica*	MG20W	(10.6)	(9)	7472*^e^*	Kim *et al.* (2015)
* Candida carpophila*	JCM9396	(10.2)	(10)	5418	R. Manabe, R. Endoh, S. Uzuhashi, G. Okada, M. Takashima and M. Ohkuma unpublished data*^d^*
* Millerozyma acaciae*	JCM10732	(11.1)	(10)	5217	R. Manabe, R. Endoh, S. Uzuhashi, G. Okada, M. Takashima and M. Ohkuma unpublished data*^d^*
* Lodderomyces elongisporus*	CBS2605^T^	(15.4)	(11)	5931	Butler *et al.* (2009)
*** Candida albicans***	SC5314	**14.3**	**2** × **8***^f^*	6207	Jones *et al.* (2004)
* Candida dubliniensis*	CD36	**14.6**	**2** × **8***^f^*	6070	Jackson *et al.* (2009)
* Candida tropicalis*	MYA3404	(14.6)	(24)	6445	Butler *et al.* (2009)
* Candida orthopsilosis*	90-125	**12.6**	**2** × **8***^f^*	5707	Riccombeni *et al.* (2012)
*** Candida parapsilosis***	CDC317	(13.1)	(24)	5843	Butler *et al.* (2009)
*** Scheffersomyces stipitis***	CBS6054	**15.4**	**8**	6026	Jeffries *et al.* (2007)
* Scheffersomyces lignosum*	JCM9837	(16.6)	(19)	6330	R. Manabe, R. Endoh, S. Uzuhashi, G. Okada, M. Takashima and M. Ohkuma unpublished data*^d^*
* Spathaspora passalidarum*	NRRL Y-27907	(13.2)	(8)	6071	Wohlbach *et al.* (2011)
* Spathaspora arborariae*	UFMG-HM19.1A^T^	(12.7)	(41)	5625	Lobo *et al.* (2014)
* Candida tanzawaensis*	NRRL Y-17324^T^	(13.1)	(16)	5895	Riley *et al.* (2016)
*** Candida (Yamadazyma) tenuis***	NRRL Y-1498	(10.7)	(61)	5533	Wohlbach *et al.* (2011)
**Metchnikowiaceae**					
* Clavispora lusitaniae*	ATCC42720	**12.1**	**8**	6116	Butler *et al.* (2009)
* Metchnikowia bicuspidata*	NRRL YB-4993	(15.1)	(48)	5851	Riley *et al.* (2016)
* Metchnikowia fructicula*	277	(26.1)	(93)	9631*^g^*	Hershkovitz *et al.* (2013)
* Candida auris*	Ci6684	(12.5)	(99)	8358*^h^*	Chatterjee *et al.* (2015)
* Candida intermedia*	JCM1607	(13.0)	(12)	5823	R. Manabe, R. Endoh, S. Uzuhashi, G. Okada, M. Takashima and M. Ohkuma unpublished data*^d^*
**Related species**					
* Babjeviella inositovora*	NRRL Y-12698^T^	(15.2)	(49)	6403	Riley *et al.* (2016)
* Cephaloascus albidus*	ATCC66658	(15.3)	(25)	6196	J. K. Magnuson unpublished data*^i^*
* Cephaloascus fragrans*	12-1022	(13.7)	(16)	5717	J. K. Magnuson unpublished data*^i^*
* Hyphopichia burtonii*	NRRL Y-1933^T^	(12.4)	(27)	6002	Riley *et al.* (2016)
* Candida homilentoma*	JCM1507	(12.2)	(8)	5661	R. Manabe, R. Endoh, S. Uzuhashi, G. Okada, M. Takashima and M. Ohkuma unpublished data*^d^*
* Wickerhamia fluorescens*	JCM1821	(13.2)	(20)	5676	R. Manabe, R. Endoh, S. Uzuhashi, G. Okada, M. Takashima and M. Ohkuma unpublished data*^d^*

The table lists the yeast species of indicated families under the same conditions as specified in Table 1. Strain numbers correspond to the reference indicated. Species in which several isolates have been sequenced in complete form or permanent drafts are indicated by bold type, and only one isolate is listed (the first one or the best one).

a Genome sizes are indicated in bold type when determined from complete sequences and in brackets when deduced from scaffolds in assemblies (figures are haploid equivalent in case of known diploid strains). Sizes ignore rDNA, mtDNA, and plasmids.

b Numbers of chromosomes are indicated in bold type when known. Figures in brackets correspond to numbers of scaffolds in assemblies.

c Total numbers of predicted protein-coding genes (CDS) are taken from original publications or subsequent annotations, as most appropriate.

d Unpublished data taken from the National BioRecource Project (www.jcm.riken.jp/cgi-bin/nbrp/nbrp_list.cgi).

e Automated prediction.

f Diploids.

g Number of *unigenes* determined from RNA sequencing, genome sequence unpublished, data taken from GenBank: ANFW000000000.2. Assembly size suggests hybrid genome.

h Dubious number, average CDS size (1024 bp) is significantly shorter than that of other yeast genomes (∼1500 bp), suggesting sequencing errors (frameshifts).

i Unpublished data taken from http://genome.jgi.doe.gov (nonpublic).

**Table 3:** Major reference genomes of the methylotroph and basal lineages

Species	Strain	Genom*e* size (Mb)*^a^*	No. of chromosomes*^b^*	Total no. of CDS*^c^*	Reference or source
**Methylotrophs**					
*** *Phaffomycetaceae**					
* Komagataella phaffii*	CBS 7435	**9.4**	**4**	5325	Küberl *et al.* (2011)
* Candida sorboxylosa*	JCM1536	(10.7)	(37)	4724	R. Manabe, R. Endoh, S. Uzuhashi, G. Okada, M. Takashima and M. Ohkuma unpublished data*^d^*
*** *Pichiaceae** and related **Saccharomycetales *incertae sedis***					
*** Dekkera bruxellensis***	CBS 2499	**13.4**	—	5208	Piškur *et al.*, (2012)
*** Dekkera anomala***	YV396	(12.9)	(30)	5241	Y. Vervoort, B. Herrera-Malaver, S. Mertens, V. Guadalupe Medina, J. Duitama, L. Michiels, G. Derdelinckx, K. Voordeckers and K. J. Verstrepen unpublished data*^e^*
* Dekkera naardensis*	CBS7540	(11.3)	(76)	—	H. Jiang, unpublished data*^f^*
* Nakazawaea peltata*	JCM9829	(11.7)	(11)	5620	R. Manabe, R. Endoh, S. Uzuhashi, G. Okada, M. Takashima and M. Ohkuma unpublished data*^d^*
* Pichia membranifaciens*	NRRL Y-2248^T^	(11.6)	(11)	5546	Riley *et al.* (2016)
* Kuraishia capsulata*	CBS 1993^T^	**11.4**	**7**	6029	Morales *et al.* (2013)
*** Ogataea polymorpha***	NCYC495 leu1.1	(9.0)	**7**	5177	Riley *et al.* (2016)
* Ogataea parapolymorpha*	DL-1	**9.1**	**7**	5325	Ravin *et al.* (2013)
* Ogataea methanolica*	JCM10240	(15.1)	(32)	6063	R. Manabe, R. Endoh, S. Uzuhashi, G. Okada, M. Takashima and M. Ohkuma unpublished data*^d^*
* Candida boidinii*	JCM9604	(19.4)	(32)	6053	R. Manabe, R. Endoh, S. Uzuhashi, G. Okada, M. Takashima and M. Ohkuma, unpublished data*^d^*
* Ambrosiozyma kashinagacola*	JCM15019	(12.3)	(23)	5787	R. Manabe, R. Endoh, S. Uzuhashi, G. Okada, M. Takashima and M. Ohkuma, unpublished data*^d^*
* Candida succiphila*	JCM9445	(12.1)	(22)	5455	R. Manabe, R. Endoh, S. Uzuhashi, G. Okada, M. Takashima and M. Ohkuma, unpublished data*^d^*
* Candida arabinofermentans*	NRRL YB-2248^T^	**(13.2)**	(62)	5861	Riley *et al.* (2016)
*** Pachysolen tannophilus***	CBS 4044	(12.2)	(34)	5546	Liu *et al.* (2012)
**Basal lineages**					
*** *Dipodascaceae, Trichomonascaceae**, and related **Saccharomycetales *incertae sedis***					
* Geotrichum candidum*	CLIB918	**24.2**	(134)*^g^*	6804	Morel *et al.* (2015)
*** Yarrowia lipolytica***	CLIB122 (E150)	**20.6**	**6**	6582	Dujon *et al.* (2004)
* Yarrowia keelungensis*	JCM14894	(21.8)	(41)	6618	R. Manabe, R. Endoh, S. Uzuhashi, G. Okada, M. Takashima and M. Ohkuma unpublished data*^d^*
* Yarrowia deformans*	JCM1694	(20.9)	(44)	6704	R. Manabe, R. Endoh, S. Uzuhashi, G. Okada, M. Takashima and M. Ohkuma unpublished data*^d^*
* Starmerella bombicola*	JCM9596	(9.6)	(16)	4887	R. Manabe, R. Endoh, S. Uzuhashi, G. Okada, M. Takashima and M. Ohkuma unpublished data*^d^*
* Starmerella (Candida) apicola*	NRRL Y-50540	(9.8)	(40)	3818*^h^*	Vega-Alvarado *et al.* (2015)
* Sporopachydermia quercuum*	JCM9486	(16.4)	(15)	5992	R. Manabe, R. Endoh, S. Uzuhashi, G. Okada, M. Takashima and M. Ohkuma unpublished data*^d^*
* Blastobotrys adeninivorans*	LS3	**11.8**	**4**	6150	Kunze *et al.* (2014)
* Blastobotrys attinorum*	NRRL Y27639	(14.0)	(14)	6184	J. K. Magnuson unpublished data*^i^*
* Trichomonascus petasosporus*	NRRL YB2093	(14.5)	(79)	6567	J. K. Magnuson unpublished data*^i^*
* Sugiyamaella lignohabitans*	CBS10342	**16.0**	**4**	6820	Bellasio *et al.* (2016)
* Sugiyamaella americana*	NRRL YB2067	(16.5)	(48)	6288	J. K. Magnuson unpublished data*^i^*
* Wickerhamiella domercqiae*	JCM9478	**8.5**	**4**	4928	R. Manabe, R. Endoh, S. Uzuhashi, G. Okada, M. Takashima and M. Ohkuma unpublished data*^d^*
* Zygoascus hellenicus*	Y7136	(12.2)	(11)	5430	R. Manabe, R. Endoh, S. Uzuhashi, G. Okada, M. Takashima and M. Ohkuma unpublished data*^d^*
* Candida infanticola*	DS02	(8.1)	(22)	—	H. Lee, C. Han, G. Park, W. Jeon, H. Lee and J. Ahn. unpublished data*^j^*
* Nadsonia fulvescens* var. *elongata*	DSM6958	(13.7)	(20)	5657	Riley *et al.* (2016)
* Tortispora caseinolytica*	NRRL Y-17796^T^	**9.2**	**6**	4657	Riley *et al.* (2016)
**Early branching***^k^*					
*** *Ascoideaceae**					
* Ascoidea rubescens*	NRRL Y-17699^T^	(17.5)	(63)	6802	Riley *et al.* (2016)
* Ascoidea asiatica*	JCM7603	(20.3)	(71)	7694	R. Manabe, R. Endoh, S. Uzuhashi, G. Okada, M. Takashima and M. Ohkuma unpublished data*^d^*
*** *Lipomycetaceae**					
* Lipomyces starkeyi*	NRRL Y-11557	(21.3)	(117)*^g^*	8192	Riley *et al.* (2016)

The table lists the yeast species of indicated families under the same conditions as specified in Table 1. Strain numbers correspond to the reference indicated. Species in which several isolates have been sequenced in complete form or permanent drafts are indicated by bold type, and only one isolate is listed (the first one or the best one).

a Genome sizes are indicated in bold type when determined from complete sequences and in brackets when deduced from scaffolds in assemblies (figures are haploid equivalent in case of known diploid strains). Sizes ignore rDNA, mtDNA, and plasmids.

b Numbers of chromosomes are indicated in bold type when known. Figures in brackets correspond to numbers of scaffolds in assemblies.

c Total numbers of predicted protein-coding genes (CDS) are taken from original publications or subsequent annotations, as most appropriate. Data not found indicated by —.

d Unpublished data taken from the National BioRecource Project (www.jcm.riken.jp/cgi-bin/nbrp/nbrp_list.cgi).

e Unpublished data taken from GenBank: LCTY00000000.1.

f Unpublished data taken from GenBank: MDSA000000000.1.

g *G. candidum* and *L. starkeyi* are listed here despite their large number of scaffolds because each genome represents the only reference available, so far, in its respective lineage.

h Automated prediction based on comparisons to the CTG clade.

i Unpublished data taken from http://genome.jgi.doe.gov (nonpublic).

j Unpublished data taken from GenBank: LWLF00000000.1.

k These are listed here for convenience and are the few sequenced yeast species not included in the four major subgroups of Saccharomycotina defined in this review. See Shen *et al.* (2016) for a recent genome-based phylogenetic reconstruction. The Lipomycetaceae family is basal to all Saccharomycotina.

### Four major genomic architectures within the Saccharomycotina

**Figure 6:**
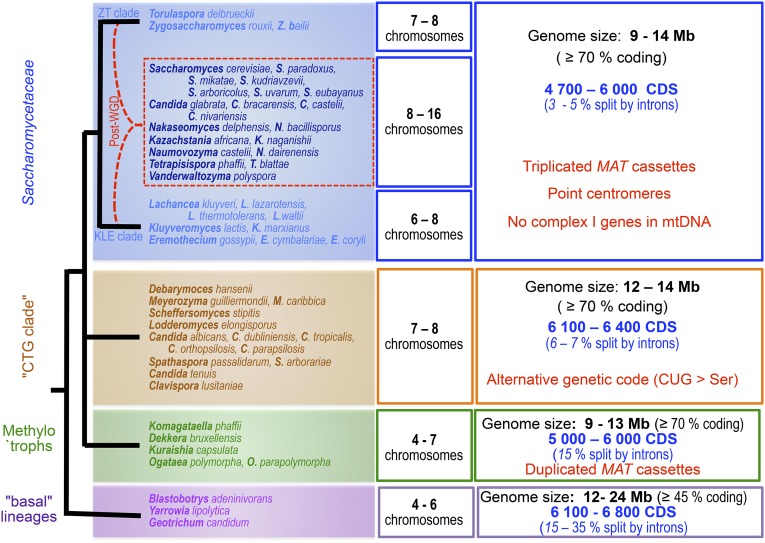
The four major subgroups of Saccharomycotina as defined from genome architectures. The major species whose genomes served to define the subgroups are indicated (see Table 1, Table 2, and Table 3 for details). Tree topology based on Kurtzman *et al.* (2011). For more recent global phylogenies, refer to Shen *et al.* (2016). Most significant properties of each subgroup are summarized in boxes on the right (see text). Red dotted lines symbolize hybridization between members of the ZT and KLE clades of Saccharomycetaceae prior to WGD (Marcet-Houben and Gabaldón 2015; Wolfe 2015).

From presently available genome sequences, four major subgroups can be recognized within the Saccharomycotina subphylum of yeasts: (i) the Saccharomycetaceae, by far the most extensively studied family; (ii) the “CTG clade,” a diversified subgroup made of yeast species using an alternative genetic code; (iii) the “methylotroph clade,” exemplified by a few recently sequenced yeasts bearing novel signatures; and (iv) several species belonging to distinct and probably distant lineages altogether regarded as “basal” to the Saccharomycotina subphylum but actually very heterogeneous. The four subgroups differ from one another by the presence/absence of specific genomic signatures (Figure 6) and are clearly separable from one another by overall proteome comparisons. Additional species may eventually illustrate novel genomic architectures or interesting evolutionary intermediates between the four subgroups, but data are presently too dispersed for definitive conclusions. The most interesting ones so far are represented by members of the Ascoidaceae and Lipomycetaceae families (see Table 3), which represent early branching lineages at the basis of the Saccharomycetaceae and all Saccharomycotina, respectively (Shen *et al.* 2016).

Genomes of the Saccharomycetaceae family (Table 1) harbor several signatures that distinguish them from all other subgroups: the presence of point centromeres, the triplication of mating loci, and the absence of genes for complex I subunits of the respiratory chain in their mitochondrial DNA (mtDNA). In addition, they generally contain a single rDNA locus either internal to a chromosome arm or, less frequently, located in subtelomeric regions as a result of independent translocation events during the evolution of the family (Proux-Wera *et al.* 2013). The rDNA locus is made of large tandem arrays of the 35S precursor transcript of ribosomal RNAs (rRNAs), like in all eukaryotes, but also includes the 5S RNA gene, most often in opposite orientation and sometimes duplicated (Bergeron and Drouin 2008). Remarkably, almost all yeasts of this family use the bacterial mode of tRNA decoding instead of the eukaryotic mode for the arginine CGN and the leucine CUN codon families (Marck *et al.* 2006; Grosjean *et al.* 2010; see below). Genomes vary in size between ∼9 and 14 Mb (haploid equivalent, ignoring the rDNA locus, mitochondria, and plasmids) but are always highly compact (∼65–75% coding) and contain ∼4500–5900 protein-coding genes with very few spliceosomal introns [3–5% of coding DNA sequences (CDS) are split]. This family is the most extensively studied, all known genera having now at least one representative fully sequenced with the sole exception of *Zygotorulaspora* and *Cyniclomyces* (the latter is basal to the family). The 2-μm family of self-replicating plasmids, initially discovered in some isolates of *S. cerevisiae* (Guerineau *et al.* 1971), seems to be circumscribed to this family where it was found in several genera (Blaisonneau *et al.* 1997; Strope *et al.* 2015a). However, plasmids in general have unfortunately not attracted sufficient attention in recent genomic works to have a precise image of their evolutionary distribution.

By contrast, sequenced members of the CTG clade (Table 2) have regional centromeres (∼3–4 kb long), often made of remnants of mobile elements, generally poorer in GC than average genomes and lacking conserved sequence motif. They have typically only one mating-type locus. Their moderately larger genomes (∼10–16 Mb, same definition as above) are also highly compact (∼55–70% coding) with ∼5600–6400 protein-coding genes and few spliceosomal introns (6–7% of CDS are split). Their major distinctive signature, relative to other subgroups, is the usage of the alternative genetic code in which the CUG codon is used to specify serine instead of leucine (or in addition to it), due to an additional tRNA species (Santos *et al.* 2011). Their mitochondrial genomes contain the seven genes encoding subunits of complex I of the respiratory chain. The different lineages in this large and heterogeneous clade have been unequally studied so far, and numerous genera have not yet been explored at the genomic level.

A smaller number of species have been sequenced so far in the methylotroph subgroup (Table 3). They share common signatures with the CTG clade (regional centromeres, high coding density, and the complete set of genes in mtDNA) except for their usage of the universal genetic code and moderately smaller genomes (∼9–13 Mb). They also exhibit a slightly higher number of spliceosomal introns (10–15% of CDS are split). Some species of this subgroup (*K. phaffii*, *Ogataea polymorpha*, and *Ogataea parapolymorpha*) have duplicated mating loci, and haploid cells undergo mating-type switching (Hanson *et al.* 2014; Maekawa and Kaneko 2014; see below). Methylotroph yeasts appear as evolutionary intermediates between Saccharomycetaceae and basal lineages by their eukaryotic mode of tRNA decoding for the leucine CUN codon family, and bacterial mode for the arginine CGN codon family (Morales *et al.* 2013). Although associated to this subgroup (Kurtzman *et al.* 2011), *K. phaffii* shares common genomic signatures with basal lineages such as the eukaryotic mode of tRNA decoding for both codon families and the dispersion of 5S RNA gene copies in the genome independently of the rDNA locus.

Basal lineages of the Saccharomycotina subphylum (Table 3) have long been represented solely by *Y. lipolytica*, whose genome exhibits a number of important differences with the three previous subgroups, such as its significantly larger size (20.5 Mb), lower compactness (46% coding), multiple rDNA loci located in subtelomeric regions, and dispersed 5S RNA gene (116 copies of this gene are distributed across the genome) like in most Pezizomycotina and Taphrinomycotina (Bergeron and Drouin 2008). With nearly 6600 protein-coding genes, 14% of which are split, it also possesses the largest protein repertoire. Additional species of *Yarrowia* that are now sequenced (see Table 3) confirm these trends. Historically, the second genome sequenced within the basal lineages was *Blastobotrys adeninivorans* (Kunze *et al.* 2014). It shares 49% of conserved microsynteny with *Y. lipolytica*, indicating a distant but still recognizable common ancestry. But its small size (12 Mb), high compactness (74% coding), and the presence of a single rDNA locus internal to a chromosome arm are more reminiscent of the evolved subgroups of Saccharomycotina. It has, however, a high number (∼6150) of protein-coding genes, 11% of which are split by introns. The genome of *Geotrichum candidum* (Morel *et al.* 2015) provided another example of the diversity of the basal lineages and the long evolutionary distances separating them. With a size (24.2 Mb), compactness (45% coding), and gene number (∼6800) comparable to *Yarrowia*, it is remarkably intron rich (35% of CDS are split) for a Saccharomycotina. Similar size (16 Mb), compactness (50% coding), and gene number (6820) were also found for *Sugiyamaella lignohabitans* (Bellasio *et al.* 2016), but only 5% of its protein-coding genes are split by introns. The recently published genomes of *Nadsonia fulvescens* and *Tortispora caseinolytica* (Riley *et al.* 2016) are comparable to *G. candidum* for their high numbers of introns, but are much smaller in size (13.7 and 9.2 Mb) and gene number. The genome of *Lipomyces starkeyi* (Riley *et al.* 2016), representative of the most basal lineage to all Saccharomycotina, is extremely intron rich with an average of three introns per protein-coding gene. The other sequences of the basal lineages reported in Table 3 (mostly unpublished) have not yet been analyzed in detail. *S. lignohabitans*, *N. fulvescens*, *T. caseinolytica*, and *L. starkeyi* have dispersed 5S RNA genes, like *Y. lipolytica*.

### Origin of the distinctive features of Saccharomycotina genomes

#### Introns

The limited number of spliceosomal introns is one of the most universal signatures of Saccharomycotina genomes (Bon *et al.* 2003; Neuvéglise *et al.* 2011). It contrasts with their intron-rich fungal ancestors (Stajich *et al.* 2007). If some members of the basal lineages of Saccharomycotina have more than a third of their CDS split by introns (an already low figure), regular figures for other yeasts are 3–5 times smaller (methylotrophs and some members of the basal lineages) or even 10 times smaller (Saccharomycetaceae). This scarcity is specific for introns of Pol-II transcripts (splicesomal introns). Yeast nuclear tRNA genes have introns in comparable numbers to other eukaryotes (with only few exceptions, see Morales *et al.* 2013), and group-I and group-II introns are frequent in yeast mitochondrial genes (see below).

The reason for the severe reduction of the number of spliceosomal introns in budding yeasts is not completely understood. An important loss may have occurred during the early stages of their evolution, perhaps associated to the loss of RNA interference (RNAi) (see below), but a tendency to additional losses seems to have continued in the different lineages. In the Saccharomycetaceae family, most introns appear to have existed in the last common ancestor and then to have been differentially lost during speciation. The overall rate of intron loss exceeds that of intron gain by two orders of magnitude, even if important quantitative differences exist in the various branches (Hooks *et al.* 2014). The mechanism by which intron-less genes are formed is not directly demonstrated, but can be imagined. The possible reinsertion of reversed-transcribed mature messenger RNAs (mRNAs) has been evoked many years ago for *S. cerevisiae* (Fink 1987), consistent with its efficient homologous recombination machinery, with the presence of numerous type-I transposable elements (encoding reverse transcriptases), and with the fact that the majority of its rare introns are located in the 5′ part of CDS. But active type-I transposable elements are not uniformly present in all yeast lineages, and the role of microhomology or nonhomologous end joining mechanisms has also been recently considered (Hooks *et al.* 2014).

Whatever the causes, the reduced prevalence of introns during yeast genome evolution has been accompanied by an increased standardization of the 5′ splice sites and branch points compared to other eukaryotes, with strong consensus sequences in both cases (6- and 7-nt long, respectively) that have only moderately evolved across the entire Saccharomycotina spectrum (Neuvéglise *et al.* 2011). Note that only U2-type introns exist because the U12 minor spliceosome has been lost in all Ascomycota (Russell *et al.* 2006). Similarly, the distance between the 3′ splice sites and branch points is also often reduced, probably by action of the splicing factor *U2AF1*, because longer distances are observed in species that have lost this factor.

The reduction of intron number in yeast genome evolution may also be correlated with the fact that, in *S. cerevisiae*, a majority of introns can be experimentally removed with no or only minor consequences for cellular fitness (Parenteau *et al.* 2008). This is not true, however, for all introns, probably explaining why a complex splicing machinery has been conserved. For example, expression of genes encoding ribosomal proteins, where introns have been frequently preserved, is affected by the presence of introns (Parenteau *et al.* 2011). Similarly, the role of introns is important for genes involved in mating (Müller *et al.* 2007). Finally, a few introns encode essential small nucleolar RNA (snoRNA) molecules and, as expected, they have been preserved during evolution (Souciet *et al.* 2009; Morales *et al.* 2013).

#### Centromeres

**Figure 7:**
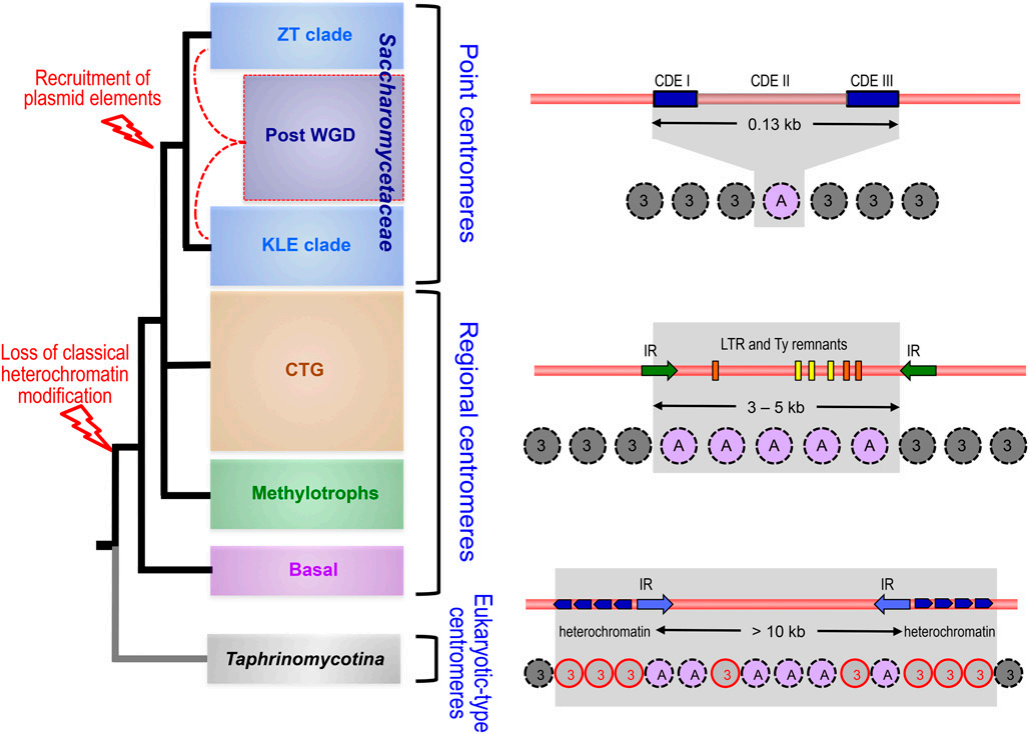
Evolution of centromeres. Left: tree topology of major subgroups of Saccharomycotina derived from Figure 6 with major evolutionary events (red lightning bolts) relative to centromeres. Taphrinomycotina used as outgroup. The loss of classical heterochromatin modification occurred very early in Saccharomycotina after the branching (not shown here) of *L. starkeyi* (Riley *et al.* 2016). Right: Centromeres (gray shade) are symbolized on chromosomes (red lines) by their characteristic elements (boxes and arrows). Symbolized below are nucleosomes (circles) of corresponding regions. Dark gray background, normal histone H3; void red circles, classical heterochromatin modifications of histone H3; purple background, CENP-A centromeric histone H3 variant (A). Adapted from Ishii (2009). Structures vary between species for regional centromeres (Chatterjee *et al.* 2016; Coughlan *et al.* 2016). IR, inverted repeats.

The presence of point centromeres, originally discovered in *S. cerevisiae* from their ability to properly partition circular plasmids during cellular divisions (Clark and Carbon 1980), is a specific landmark of the Saccharomycetaceae family (Meraldi *et al.* 2006) that raises the question of their origin (Figure 7). These short structures, made of two sequence motifs (CDE I and CDE III) separated from each other by a short AT-rich interval (CDEII), contrast with the 3- to 5-kb-long regional centromeres observed in the CTG clade (Sanyal *et al.* 2004; Padmanabhan *et al.* 2008; Kapoor *et al.* 2015; Chatterjee *et al.* 2016), in *Y. lipolytica* (Vernis *et al.* 1997, 2001; Lynch *et al.* 2010), and in the methylotrophs (Morales *et al.* 2013; Coughlan *et al.* 2016), and which are made of variable sequences often associated to clustered traces of mobile elements or flanked by inverted repeats. Regional centromeres of Saccharomycotina yeasts are reminiscent of the epigenetic centromeres of multicellular eukaryotes made of large arrays of DNA lacking sequence specificity (Brown and O’Neill 2014), but are not identical to them. Compared to *S. pombe* centromeres, whose central region made of CenH3 nucleosomes (the variant form of histone H3 essential for centromere function) is flanked by heterochromatin like in most eukaryotes, the CenH3 nucleosomes of the regional centromeres of *C. albicans* or *C. lusitaniae* are not embedded in heterochromatin (Sanyal *et al.* 2004; Kapoor *et al.* 2015). This property may facilitate their neoformation (Ketel *et al.* 2009), although the phenomenon has also been observed for large epigenetic centromeres.

It has been proposed that the loss of RNAi and of classical heterochromatin in Saccharomycotina (see below) was at the origin of the evolution of their centromeres (Baum *et al.* 2006; Padmanabhan *et al.* 2008; Ketel *et al.* 2009; Malik and Henikoff 2009). The emergence of point centromeres in the Sacharomycetaceae, in which the CenH3 protein is conserved, must have taken place by recruiting novel components such as Ndc10p and Ctf13p to bind the evolutionarily conserved kinetochore machinery to the CDEIII motif, forming the CBF3 complex (Meraldi *et al.* 2006). Malik and Henikoff (2009) have argued that these two genes, which have no homologs in other organisms, might have originated from the capture of the *REP1* and *REP2* genes of the 2-μm plasmids specifically found in the Saccharomycetaceae yeasts (Blaisonneau *et al.* 1997). According to this view, the first point centromeres may have derived from the chromosomal integration of the *cis*-acting STB locus of these plasmids, hence alleviating the need of the ancestral regional centromeres. How this initial structure spread to all chromosomes remains an open question but, once established, point centromeres have been highly conserved in Saccharomycetaceae chromosomes as judged from the conservation of their flanking genes. This evolutionary process, however, has not been unique as revealed by the new type of point centromeres recently discovered at different chromosomal locations in *Naumovozyma castelli* and *Naumovozyma dairenensis* (Kobayashi *et al.* 2015). The fact that the overexpression of *CSE4* (encoding the CenH3 protein) reveals new centromere-like regions (CLR) in the genome of *S. cerevisiae* suggests a possible mechanism at the origin of novel point centromeres (Lefrancois *et al.* 2013). CLR are similar in size to point centromeres but lack sequence specificity.

#### Mating cassettes and mating-type switching

**Figure 8:**
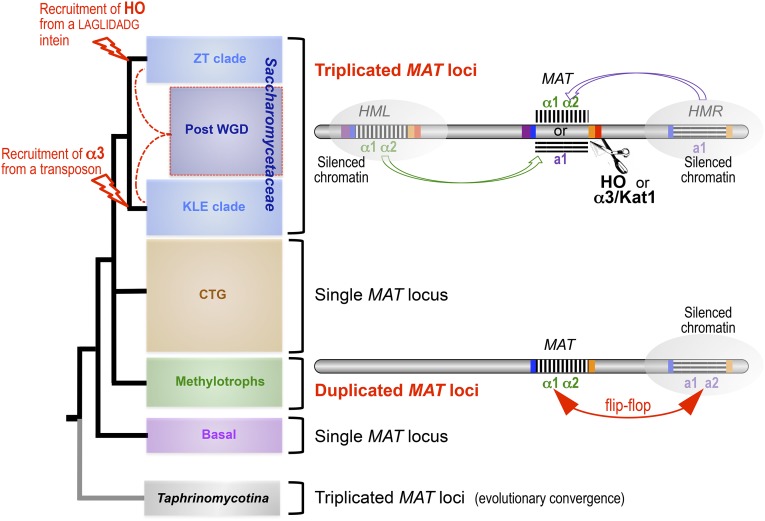
Evolution of mating-type switching. Left: same as Figure 7. Red lightning bolts refer to capture of site-specific nucleases. Right: *MAT* cassettes (colored boxes) are symbolized on chromosomes (gray bars) with *MAT***a** (horizontal black stripes) or *MAT*α (vertical black stripes) idiomorphs. Silenced cassettes are shaded. Shown are the topologies observed in *S. cerevisiae* (for the Saccharomycetaceae) and *K. phaffii* (for the methylotrophs), but significant variations are found in both subgroups (see text). The α3 and Kat1 transposases of *K. lactis* are shown acting at the same place as the HO endonuclease of *S. cerevisiae*, despite important mechanistic differences.

Mating-type switching by the conversion of one idiomorph to its opposite at the *MAT* locus during mitotic cycles of yeasts has retained considerable attention during the last decades (Heitman *et al.* 2013). It is a characteristic feature of yeasts that differentiates them from their multicellular fungal ancestors. In *S. cerevisiae* and *S. pombe*, the molecular mechanisms at the basis of the phenomenon have been precisely elucidated and they extensively differ (Haber 2012; Klar *et al.* 2014). Despite the fact that these mechanisms are inherently destructive for genomes (Gordon *et al.* 2011), mating-type switching has emerged independently several times during yeast evolution, suggesting some selective advantage (Figure 8). One of them might be that switching facilitates the finding of a mating partner of the same species in harsh environmental conditions (Hanson *et al.* 2014). Another one might be that, by forming homozygous diploids, switching alleviates the risk of dissociation of favorable allelic combinations at the next meiosis, such combinations resulting from insidious selection during the long phases of clonal propagation that are so characteristic of yeasts.

In *S. cerevisiae*, mating-type switching involves two extra copies of the *MAT* locus (designated *HML* and *HMR* cassettes, respectively), located close to telomeres on the same chromosome and silenced by a combination of the SIR1–4 proteins. Note that the common histone modification (H3K9me), characteristic of silent chromatin in most eukaryotes, is absent in Saccharomycotina due to their original loss of the *CLR4* and *SWI6/HP1* genes (Hickman *et al.* 2011). Such a triplication is another intrinsic landmark of genomes of the Saccharomycetaceae family not found in other Saccharomycotina (Figure 8). Members of the CTG clade as well as *Y. lipolytica* have only one *MAT* locus per haploid genome, designated *MTL*, and do not switch mating type. *MTL* bears either the *MAT***a** or *MAT*α idiomorphs with some rare cases of mixing of both (Dujon *et al.* 2004; Butler *et al.* 2009). In the heterothallic members of the methylotrophic subgroup capable of switching, the *MAT* locus is duplicated (see below). Triplication of the *MAT* locus also occurred in *S. pombe* and other *Schizosaccharomyces* species, but this represents an evolutionary convergence (Klar *et al.* 2014), not an ancestral feature. During evolution of the Saccharomycetaceae family, the triplication has received secondary modifications such as the loss of one or two cassettes in some species with the concomitant loss of switching or even sex (Fabre *et al.* 2005; Müller *et al.* 2007; Wolfe *et al.* 2015), or the expansion to a fourth locus in members of the *Eremothecium* clade (Dietrich *et al.* 2013).

In *S. cerevisiae*, the *HML* cassette bears copies of the *MAT*α*1* and *MAT*α*2* genes, and the *HMR* cassette has a copy of the *MAT***a***1* gene (encoding a homeodomain protein that drives meiosis and sporulation). The *MAT***a***2* gene, encoding a transcriptional activator belonging to the HMG family conserved in most yeast species, has been lost in the post-WGD species of the Saccharomycetaceae family but is present in members of the ZT and KLE clades (see below and Table 1). In *S. cerevisiae*, the switching mechanism requires (i) a specific double-strand endonuclease (*HO*), cleaving DNA within the active *MAT* locus while leaving intact the silenced copies; (ii) a double-strand break repair machinery using the sequence homology of the flanking regions (designated X and Z); and (iii) a donor preference enhancer (RE) to ensure repair using the appropriate silent cassette (Haber 2012). The *HO* gene is also found in *Z. rouxii*, *Torulaspora delbrucki* (ZT clade), and almost all post-WGD species of Saccharomycetaceae (lost in *Kazachstania africana*), but it is not found in species of the KLE clade of Saccharomycetaceae. It encodes an endonuclease belonging to the LAGLIDADG family of intein-related proteins that has probably been recruited for mating-type switching in the ancestor of the ZT clade and transmitted to the post-WGD species, or, perhaps, recruited in the ancestor of the entire family but then lost in members of the KLE clade (no species has a complete *HO* gene). The presence of sequences similar to *HO* in some of these last species (Fabre *et al.* 2005) corresponds to relics of an ancestral gene of the same LAGLIDADG family that may or may not have been involved in mating-type switching.

The entirely different switching mechanism discovered in *K. lactis* (Barsoum *et al.* 2010; Rajaei *et al.* 2014) indicates the independent recruitment of a completely different machinery in this lineage. In this case, the *MAT*α to *MAT***a** switching events and the reverse *MAT***a** to *MAT*α events involve two domesticated transposases, α3 and Kat1, respectively. α3 is encoded by the *MAT*α*3* gene located next to the *MAT*α locus and shares homology to the MULE family of transposable elements. Kat1 is the product of the *KAT1* gene containing a programmed −1 frameshift and shares homology to the hAT family of transposable elements. Both are under the transcriptional control of the *MTS1*/*RME1* gene product, induced by nutrient limitation. In *MAT*α strains, binding of Mts1 to sites close to the *MAT*α*3* gene stimulates the excision of the MULE, inducing *MAT*α to *MAT***a** switching to repair the chromosome break. In *MAT***a** strains, Kat1 generates two hairpin-capped, double-strand breaks at the fossil imprints of an ancient transposon located within the *MAT***a** locus; inducing *MAT***a** to *MAT*α switching to repair the chromosome break. Within the KLE clade, the *KAT1* gene is specific to the *Kluyveromyces* species (absent from *L. thermotolerans* and *E. gossypii*), suggesting a domestication event of the transposon at the origin of this genus (Rajaei *et al.* 2014).

The mechanisms of mating-type switching in some methylotrophic yeasts may help us understand the origin of the complex three-locus system that evolved in the Saccharomycetaceae (Hanson *et al.* 2014; Maekawa and Kaneko 2014). In *O. polymorpha* and *K. phaffii* (see Table 3), two *MAT* loci bearing opposite idiomorphs lie in opposite orientation on the same chromosome. They are flanked by inverted repeats. One is the active locus that determines cell type, the other is silenced by its proximity to the centromere (*O. polymorpha*) or the telomere (*K. phaffii*). In both cases, switching occurs by a flip-flop mechanism that inverts the chromosomal segment between the two loci at the same time as it exchanges idiomorphs between active and silent locations. The phenomenon is induced by nutrient starvation. The same situation was recently found in *Pachysolen tannophilus* and *Ascoidea rubescens* (Riley *et al.* 2016). Whether or not this two-locus system has a common origin with the three-locus system of the Saccharomycetaceae remains an open question given the important differences between the molecular mechanisms involved. But its presence in *A. rubescens*, a member of the Ascoideaceae family, is consistent with an evolutionary intermediate between the methylotrophs and the Saccharomycetaceae.

#### tRNA decoding and alternative genetic codes

The alteration of the genetic code in *C. albicans* and related species, with the CUN codon read as serine instead of leucine, has attracted attention long (Santos and Tuite, 1995; Sugita and Nakase 1999) before being recognized as a common characteristic of >75 species of the CTG clade (Santos *et al.* 2011). Contrary to several other alterations of the code that often concern nonsense codons (the reading of UGA as tryptophan in yeast mitochondria is one such example), this sense-to-sense alteration is mediated by a Ser tRNA containing a 5′-CAG-3′anticodon (Ser-tRNA^[CAG]^), which is efficiently charged by the seryl-tRNA synthetase and occasionally charged by the leucyl-tRNA synthetase. Ratios of 97 *vs.* 3% are reported under normal conditions. This unusual tRNA appeared prior to the split between the CTG clade and other subgroups of Saccharomycotina, and must have competed for a long time with the normal Leu-tRNA^[CAG]^ for mRNA decoding before the latter was eventually lost (Yokogawa *et al.* 1992; Massey *et al.* 2003). Its formation involved three mutational steps around the anticodon, plus one in the variable loop (Santos *et al.* 2011) that must have occurred in a stepwise manner because basal representatives of this clade, such as *Metchnikowia bicuspidata* and *Babjeviella inositovora*, have only some of these changes (Riley *et al.* 2016). The CUG reassignment was associated to massive mutational change of CUG codons to UUG or UUA codons in protein-coding genes to preserve leucine in proteins, whereas new CUG codons were created at positions corresponding to serine or amino acids of similar chemical properties in other yeast proteins. It remains that the ambiguous identity of the CUG codons in these yeasts generates “statistical” proteins whose biological implications may have been important for the evolution of the CTG clade.

Alteration of the CUG codon significance in the nucleus is not unique to yeasts of the CTG clade. It has occurred several times during yeast evolution as a result of distinct mechanisms (Mühlhausen *et al.* 2016). In *P. tannophilus*, representing a basal lineage to the methylotroph clade, as well as in *A. rubescens*, a member of the Ascoidaceae family, the CUG codon specifies alanine instead of leucine or serine (Riley *et al.* 2016). In *P. tannophilus*, reassignment followed tRNA loss (Mühlhausen *et al.* 2016). Several different sense-to-sense codon reassignments are also observed in the mitochondrial genetic code of the Saccharomycetaceae yeasts (Ling *et al.* 2014, for a recent overview).

Alteration of tRNA molecules was not rare in the evolution of Saccharomycotina yeasts, and the total number of genes encoding them varied to a large extent between ∼80 for several members of the methylotroph subgroup (Morales *et al.* 2013) to >500 for *Y. lipolytica* (Dujon *et al.* 2004; Marck *et al.* 2006). Whereas the global degree of redundancy is generally conserved (some tRNA species are always encoded by multiple genes, others by few), the repertoire of tRNA species has evolved between the different yeast lineages. This is particularly remarkable for the leucine and arginine four-codon families. When *Y. lipolytica* and *K. phaffii* use the eukaryotic mode of tRNA decoding for both CUN and CGN codon families, as expected of their fungal ancestry, most members of the Saccharomycetaceae family use a bacterial mode for the same codons (the difference corresponds to the nucleotide at position 34 which is used to interpret the wobble position of the codons; Marck *et al.* 2006). Interestingly, members of the methylotroph subgroup use the ancestral eukaryotic mode for the Leu-CUN family, but the bacterial mode for the Arg-CGN family. As members of the CTG clade also use the bacterial mode of decoding for the Arg-CGN family, the transition from the eukaryotic mode to the bacterial mode for this family must have occurred very early during the evolution of the Saccharomycotina, immediately after the separation of the basal lineages; whereas the transition for the Leu-CUN family seems to be specific to the Saccharomycetaceae.

#### RNAi machinery

It is sometimes argued that, owing to the power of its genetic tools, RNAi would probably have been discovered earlier if it had existed in *S. cerevisiae*. But it does not. And the same is true for most Saccharomycotina, but not all. The evolution of this important eukaryotic function in budding yeasts appears complex if one judges from the patchy distributions of the *Dicer* and *Argonaute* genes in the Saccharomycetaceae family (Wolfe *et al.* 2015), a phenomenon also observed in other fungi where the variable numbers of these two genes suggest recent duplications and losses (Hu *et al.* 2013).

The existence of RNAi in Saccharomycotina was discovered by analysis of *C. albicans*, the most intensively studied pathogen of the CTG clade; and *N. castellii*, a poorly studied member of the Saccharomycetaceae family (Drinnenberg *et al.* 2009). Contrary to *S. cerevisiae*, these two yeasts are able to generate small interfering RNAs, mostly corresponding to transposable elements and subtelomeric Y′ elements, using noncanonical *Dicer* proteins. They also have an *Argonaute* gene. The same is true for *V. polyspora* and several other post-WGD genera of Saccharomycetaceae, but not for *Saccharomyces* (Wolfe *et al.* 2015). Detailed structural analysis, however, indicates that the canonical Dicer (ancestral) was probably lost at the origin of the entire Saccharomycotina lineage; the noncanonical dicer activity being reestablished after duplication of genes of the RNase-III family and separation of the rRNA-, snoRNA-, and small nuclear RNA-processing activity conserved in all yeasts (*RNT1* gene in *S. cerevisiae*) from a neo-dicer activity (*DCR1* gene in *N. castellii*) conserved in only some lineages (Bernstein *et al.* 2012a). In *C. albicans*, the *DCR1* gene also ensures the maturation of the ribosomal and spliceosomal RNAs, allowing the secondary inactivation of the *RNT1* gene (Bernstein *et al.* 2012b).

The loss of RNAi activity in some lineages of Saccharomycotina has probably greatly influenced their mode of evolution. Besides the loss of the multiple controls and regulations, it has probably played a role in their ability to host double-stranded, viral-like RNA elements such as the killer particles in *S. cerevisiae* (Wickner *et al.* 2013), as was experimentally demonstrated in *S. cerevisiae* (Drinnenberg *et al.* 2011). Similarly, the remarkable lengthening in yeasts of some peripheral branches in otherwise conserved noncoding RNA molecules (Kachouri *et al.* 2005; Mitrovich and Guthrie 2007) may be another direct consequence of this phenomenon. The telomerase RNA, for example, is >2-kb long in *C. glabrata* (Kachouri-Lafond *et al.* 2009).

#### mtDNA

Like in most eukaryotes, yeast mtDNA encodes the RNA molecules necessary to ensure an independent protein synthesis from the cytoplasm: genes for the large and small rRNAs, a set of 23–25 distinct tRNAs sufficient to read the entire genetic code (with some differences from the “universal” one) and, often, the RNA subunit of the mitochondrial RNase P. This machinery translates RNA from only a few mitochondrial genes encoding subunits of the respiratory chain (4–11 genes, depending on the species) and of the ATP synthase complex (2–3 genes), as well as one protein (Var1) involved in the late-step assembly of the small subunit of the mitochondrial ribosome. Mitochondrial ribosomes and tRNAs also translate the reading frames of group-I and group-II introns which, although optional, may equal or outnumber the above-mentioned protein-coding genes. These notions have been known for a very long time, thanks in particular to the pioneering work performed >3 decades ago on *S. cerevisiae* (reviewed in Dujon 1981). In this species, the mitochondrial genome consists of a few dozens of copies of a ∼80-kb circular mtDNA molecule containing, in addition to the above, long AT-rich intergenic regions including short GC-rich palindromes. They recombine between themselves (Dujon *et al.* 1974; Fritsch *et al.* 2014). Today, the evolution of the mitochondrial genomes of Saccharomycotina is illustrated by the complete mtDNA sequences of >80 different species (reviewed in Freel *et al.* 2015) and it shows a remarkable consistency with the four subdivisions deduced from nuclear genomes (above).

The *VAR1* gene is present in all subgroups except the CTG clade. Its presence in the basal groups indicates its ancestral origin and long-range conservation despite the fact that its sequence evolves very rapidly. The reason for the loss of *VAR1* in the CTG clade remains unclear. It may be that assembly of the mitochondrial ribosome in which all other proteins are encoded by the nucleus was modified by the alteration of the genetic code. *VAR1* has also been lost from the mtDNA of *Hanseniaspora uvarum*, but this species, a member of the Saccharomycodaceae family, has a linear mitochondrial genome extremely reduced in size (∼11 kb) (Pramateftaki *et al.* 2006). The loss of genes encoding complex-I subunits of the respiratory chain in mtDNA is a landmark of the Saccharomycetaceae genomes that differentiates them from other subgroups of Saccharomycotina in which, with only few exceptions, mtDNA always encodes seven subunits of the NADH:ubiquinone oxidoreductase complex like in most eukaryotes (reviewed in Freel *et al.* 2015). Remarkably, the same loss occurred in the *Schizosaccharomyces* genomes; another example of evolutionary convergence between the two most extensively studied yeast species. The loss of complex I was found coupled to the duplication of nuclear genes for alternative dehydrogenases, a phenomenon possibly related with the adaptation to fermentative metabolism (Marcet-Houben *et al.* 2009). Beside these important differences, several other variations occurred during the evolution of yeast mitochondrial genomes. In the *Saccharomyces*, the presence of an abnormal tRNA molecule reads the CUN codons as threonine (Dujon 1981). In *C. glabrata*, the CGN-codon family has been entirely eliminated due to the loss of the cognate tRNA molecule (Koszul *et al.* 2003). Considerable size variation of mtDNA molecules is also observed between related species, ranging from ∼20 kb in *C. glabrata* to almost 110 kb in *Nakaseomyces bacillisporus* (Bouchier *et al.* 2009). Size variation is due in part to the presence/absence of group-I and group-II introns. But it also results from the invasion of short GC-rich palindromic clusters of unclear origin associated with the expansion of large AT-rich intergenic regions (Bouchier *et al.* 2009). These elements were discovered long ago in *S. cerevisiae* (Dujon 1981), but their patchy distribution across the entire phylogeny of Saccharomycotina species (Freel *et al.* 2015) raises an interesting problem to examine.

### Tentative reconstruction of the evolution of Saccharomycotina genomes

**Figure 9:**
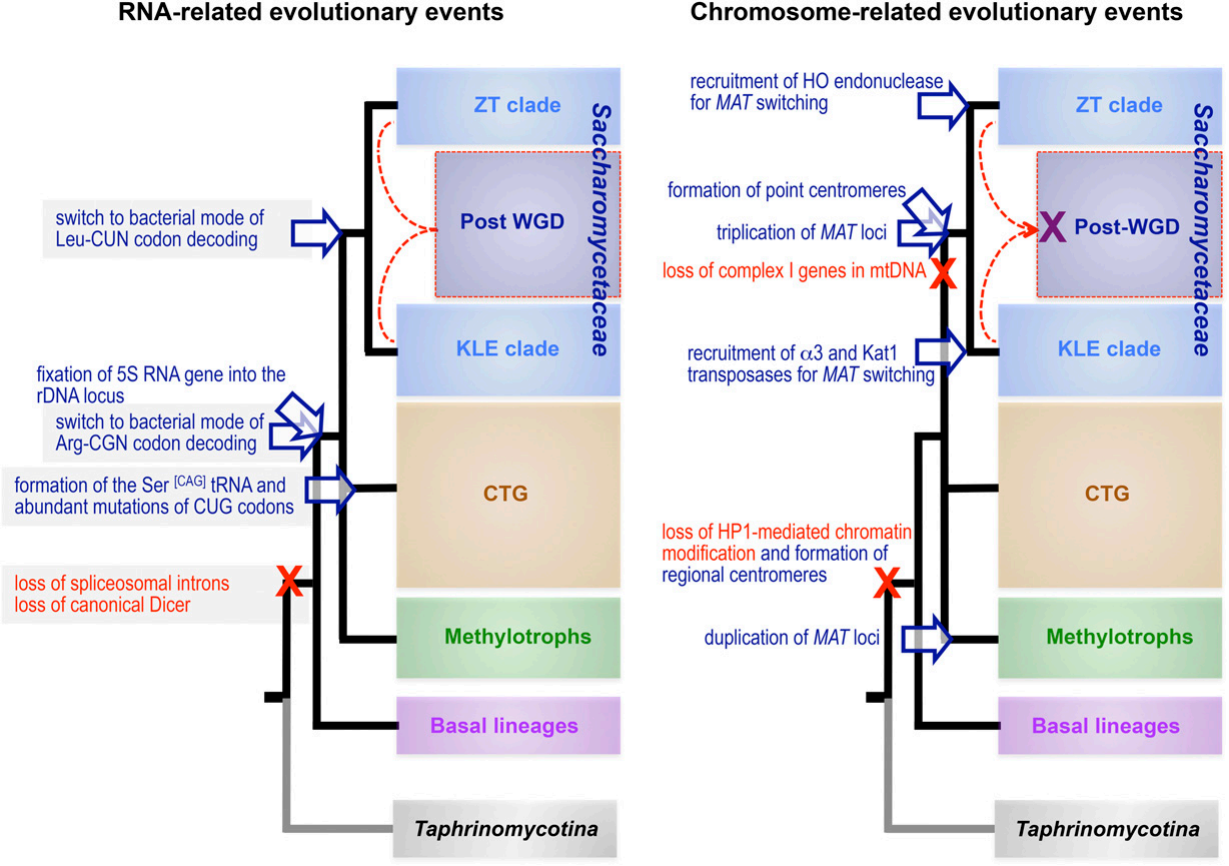
Overview of Saccharomycotina genome evolution. Evolutionary innovations (blue arrows) or losses (red crosses) attributed from parsimony to major separations between subgroups of Saccharomycotina (secondary losses of preceding innovations, not shown for simplicity). Left: RNA-related events. Note that, in some lineages, noncanonical enzymes replaced the ancestral Dicer and that the phylogenetic distribution of Dicer and Argonaute proteins in Saccharomycotina is discontinuous (see text). Right: chromosome-related events. Note that the recruitment of α3 and Kat1 transposases concerns the *Kluyveromyces*, not the entire KLE clade, and that the loss of H3K9me chromatin modification occurred after the separation of *L. starkeyi* (data not shown) from other basal lineages. Purple cross: massive post-WGD gene loss. Note that the loss of complex-I genes in mtDNA is not a chromosomal event, but its occurrence is indicated because of its association with the duplication of nuclear genes encoding alternative dehydrogenases (see text).

Considering the above elements, the important evolutionary changes separating the four major subgroups of Saccharomycotina genomes can be summarized as illustrated by Figure 9 using parsimony as a guiding principle, but keeping in mind that more complex scenarios, including multiple and reversible changes, are not excluded. As discussed above, some changes concern RNA molecules and others concern chromosome structure. Among the latter, some events correspond to the intrinsic dynamics of chromosomes in vertical lineages and others are related to horizontal exchanges such as the capture of extrachromosomal elements or interspecific hybridizations (see below). The loss of ancestral elements and functions also played a critical role in subsequent evolutionary events of the Saccharomycotina subphylum. Among those, the early losses of HP1-mediated formation of heterochromatin (Hickman *et al.* 2011; Riley *et al.* 2016) and of canonical eukaryotic RNAi (Bernstein *et al.* 2012a) were probably the two most important events. The decreasing number of spliceosomal introns and the increasing compactness of genomes are general trends that seem to have persisted throughout the evolution of the Saccharomycotina subphylum, but were already significant at its origin.

Among the RNA-related events, a fixation of the 5S RNA gene into the rDNA locus must have occurred very early, as only some members of the basal lineages have dispersed 5S RNA genes (a normal situation for eukaryotes) and no 5S gene in the rDNA locus. Most other yeasts have one (or rarely two) 5S gene(s) in the rDNA locus and no (or few) dispersed copies. But this fixation was probably reversible since some members of the CTG and methylotroph clades, such as *M. bicuspicata*, *Hyphophichia burtonii*, and *K. phaffii*, only have dispersed copies in multiple numbers (Riley *et al.* 2016). Note, however, that these species represent lateral branches to these clades. The switch to the bacterial decoding mode of the Arg-CGN codon family also occurred very early, whereas the situation appears more complex for the Leu-CUN codon family (with some reversions and multiple reassignments of the CUG codon).

Most chromosome-related events concern the Saccharomycetaceae family, but this is also where they have been most extensively studied, particularly in *S. cerevisiae*. The WGD was recognized very early (Wolfe and Shield 1997) and its origin and consequences have been extensively studied to the point that post-WGD species are overrepresented among sequenced yeasts (see Table 1). The event, suspected to have occurred from an ancestor with eight chromosomes (Gordon *et al.* 2009), was followed by extensive gene loss (Scannell *et al.* 2007a,b), while neofunctionalization or subfunctionalization of the remaining paralogous copies (ohnologs) have played an important functional role in the evolution of these yeasts. The existence in post-WGD genomes of numerous paralogs predating the WGD event was recently interpreted as an indication that the duplication event followed an interspecies hybridization event that probably occurred between a member of the ZT clade (which appears phylogenetically closer to post-WGD) and a member of the KLE clade of Saccharomycetaceae (Marcet-Houben and Gabaldón 2015; Wolfe 2015).

The triplication of the *MAT* cassettes exists in both clades (with variations in some species) but the recruitment of the HO endonuclease for mating-type switching (probably from a LAGLIDADG intein) is specific to the ZT clade and, consequently, post-WGD species. Less data are unfortunately available about the recruitment of the α3 and Kat1 transposases discovered in *K. lactis* (Barsoum *et al.* 2010; Rajaei *et al.* 2014) and about the mechanism of mating-type switching in other members of the KLE clade. How the duplication of the *MAT* cassettes in *A. rubescens* and some methylotroph yeasts (Hanson *et al.* 2014; Maekawa and Kaneko 2014; Riley *et al.* 2016) relate to the triplication in Saccharomycetaceae is not yet entirely solved, but it was inferred that mating-type switching evolved soon after the loss of the classical H3K9 chromatin modification, a very early event at the origin of all Saccharomycotina after the separation of *L. starkeyi* (Riley *et al.* 2016).

Another highly spectacular evolutionary transition at the chromosomal level is the replacement of the regional centromeres found in all Saccharomycotina, except Saccharomycetaceae, by the point centromeres specific to the latter family; a phenomenon proposed to result from the capture of genes and *cis*-acting elements from the circular 2-μm plasmids into chromosomes (Malik and Henikoff 2009). Other captures have been reported which, together with other mechanisms (below), have contributed at various levels during the evolution of Saccharomycotina.

### Molecular basis of the evolutionary dynamics of yeast genomes

Yeasts remarkably illustrate the fact that the evolution of genomes is driven by a combination of intrinsic mechanisms, operating during the successive generations in vertical lineages, and horizontal exchanges with other contemporary lineages. The latter represent either regular events, as in sexual reproduction, or accidental events as in interspecies hybridization or foreign gene capture. If the two types of phenomena are necessarily entangled in eukaryotic organisms with obligate sexual reproduction, they are clearly separable in organisms, such as yeasts, capable of unlimited clonal propagation under favorable conditions and of surviving the stresses of complex ecosystems.

#### Intrinsic genome dynamics: sequence divergence and chromosomal rearrangements

Sequence divergence between yeast genomes is always much higher than anticipated by the similarities between the species. Within single genera, two species such as *S. cerevisiae* and *S. uvarum*, for example, can differ from each other by ∼33% at the nucleotidic level. Compare this to the 1.8% separating genomes of human and chimpanzee, classified in two distinct genera! Additionally, their orthologous proteins share, on average, ∼80% aa identity, a figure similar to the average identity between human and chicken proteins (Dujon 2006). Other genera are even more diverged. In *Lachancea*, the average amino-acid identity between orthologous proteins of distinct species range from 83 to 69% (Vakirlis *et al.* 2016). In *Eremothecium*, the same measure drops to ∼60% (Wendland and Walther 2011, 2014), and even lower figures (51–53%) were found within the *Nakaseomyces* genus (Gabaldón *et al.* 2013). These high sequence divergences indicate that, contrary to what is frequently written in the literature, there is no such thing as “closely related yeasts,” except in very specific instances. In agreement with their primarily clonal mode of propagation, the distinct species of yeasts only represent the remnants of separated lineages after very high numbers of successive mitotic generations and numerous population bottlenecks.

The same conclusion can be drawn from examination of synteny. If extensive conservation of gene orders can be observed between members of some genera (see *What did We Learn from Comparative and Population Genomics of the Saccharomyces Species Complex?*), chromosomal breakpoints rapidly accumulate at greater evolutionary distances, rapidly reducing the size of conserved blocks to smaller and smaller numbers of genes. In pairwise comparisons between members of the KLE clade of Saccharomycetaceae, for example, average block sizes of only 10–20 genes are generally observed (Souciet *et al.*2009), with frequent insertion/deletion or inversion of genes within them (Byrne and Wolfe 2006). A similar situation applies for the methylotrophic yeasts (Morales *et al.* 2013; Ravin *et al.* 2013). If yeast genome maps are more stable than those of vertebrates or insects for equivalent degrees of sequence divergence (Drillon and Fischer 2011; Rolland and Dujon 2011), nearly all traces of conserved synteny disappear when members of the distinct subgroups are compared, *i.e.*, the total number of chromosomal breakpoints accumulated in genomes during their evolution approaches the number of genes. The nature and extent of chromosomal rearrangements have recently been documented in detail within the single yeast genus of *Lachancea* (Vakirlis *et al.* 2016). It was found that unbalanced rearrangements, primarily made of gene duplications and losses, outnumber the balanced ones, such as inversions and translocations, and frequently disrupt genes at breakpoints.

#### Intrinsic genome dynamics: loss, duplication, and neoformation of genes

Yeasts also differ extensively from one another by their protein repertoires, due to gene losses and gains (Souciet *et al.* 2009; Libkind *et al.* 2011; Scannell *et al.* 2011; Liti *et al.* 2013; Wendland and Walther 2014; Riley *et al.* 2016; Vakirlis *et al.* 2016). Even within each of the four subgroups defined above, core genomes are significantly limited relative to pan-genomes. This is obviously not unique to yeasts, but their genomes remarkably illustrate the importance of gene loss in evolution, as well as its compensation by gene duplication and *de novo* formation.

Yeast genomes offer numerous examples of gene loss, in agreement with the fact that only a minority of genes (∼18%) are essential for cell viability, at least under laboratory conditions as demonstrated by the systematic gene deletions in *S. cerevisiae* (Giaever and Nislow 2014). If part of this phenomenon may be explained by the presence of paralogs, numerous losses of unique genes can also be observed when comparing genomes, leading to functional losses that differentiate the lineages. Species adapted to special niches such as, for example, the human pathogen *C. glabrata* (Dujon *et al.* 2004) tend to lose more genes. Remarkably, gene loss may result in the gain of novel adaptive functions by alteration of regulatory networks (Gabaldón *et al.* 2013). Most gene losses correspond to entire deletions of the corresponding chromosome segments. Pseudogenes, exhibiting various degrees of sequence alteration, are also found but remain rare in yeasts (Lafontaine and Dujon 2010). Interestingly, functionally coordinated genes, even scattered in genomes, tend to be lost in parallel in given lineages (Hittinger *et al.* 2010; Gabaldón *et al.* 2013). The importance of gene loss in yeast genome evolution is consistent with their clonal mode of propagation.

In recent work on *G. candidum*, a member of the basal lineages of Saccharomycotina, it was observed that a significant number of its genes (263 or 3.9% of total) were “phylogenetically discordant,” *i.e.*, were absent from other Saccharomycotina but present in Pezizomycotina or other fungal lineages (Morel *et al.* 2015). Their similar patristic distance with conserved genes from the common ancestor of both subphyla indicated, however, common vertical lineage rather than recent horizontal acquisitions; leading to the conclusion that these genes had been lost in all Saccharomycotina other than *G. candidum*. They were designated as specifically retained ancestral genes (SRAG). SRAGs were also observed in the genomes of *Y. lipolytica* and *D. hansenii*, where they represent 3.6 and 2.0% of the total numbers of protein-coding genes, respectively.

Because total numbers of protein-coding genes vary within only moderate limits in yeast genomes (<1.4× in general and ∼1.1× within each subgroup; Figure 6), it follows that gene gains must roughly equilibrate losses in number over long evolutionary times. Gene gains have at least four entirely distinct origins: duplications of preexisting genes; horizontal acquisitions from other genomes; capture from nonchromosomal sources such as plasmids, viruses, or mitochondria; and, finally, *de novo* gene formation. These mechanisms are of unequal importance numerically but not biologically. Duplications, *de novo* formation, and intracellular captures from mtDNA are intrinsic mechanisms of vertical inheritance; the other mechanisms involve a horizontal dimension of inheritance. In contrast to the other mechanisms, duplications do not initially expand gene family repertoires but generate collections of paralogs, altering gene number equilibriums, upon which subsequent evolutionary changes may eventually operate. The duplications themselves result from different mechanisms, as reflected from the locations of paralogous genes in yeast genomes and by the plurimodal distributions of sequence identity between their products (Dujon *et al.* 2004; Souciet *et al.* 2009; Marcet-Houben and Gabaldón 2015). These mechanisms range from the formation of tandem gene arrays to the accidental duplication of an entire genome (above) or, more frequently, to the duplication or higher-order amplification of large chromosomal segments embedding series of adjacent genes. Tandem gene arrays are observed in all yeast genomes, albeit in different proportions (Despons *et al.* 2011), and are generally not conserved between species. Segmental amplifications are frequently observed in experiments using *S. cerevisiae* (Koszul *et al.* 2004; Schacherer *et al.* 2007b; Araya *et al.* 2010; Payen *et al.* 2014; Thierry *et al.* 2015, 2016) but leave few traces in natural yeast genomes, except in subtelomeric regions (Fairhead and Dujon 2006).

The overall result of all duplication mechanisms is such that no yeast genome is minimal. Families of two or more members with varying degrees of sequence divergence represent, in general, 30–45% of total gene numbers (Butler *et al.* 2009; Souciet *et al.* 2009). Some families correspond to very ancient duplications, conserved across enormous numbers of successive generations. Others represent the result of recent duplications that may or may not be conserved in subsequent generations. The comparison between five protoploid species of the Saccharomycetaceae family (members of the ZT and KLE clades) performed some years ago (Souciet *et al.* 2009) gave us the first indication of the kinetics involved. A little more than 100 pairs of dispersed paralogs (114) were completely conserved between *Z. zouxii*, *L. kluyveri*, *L. thermotolerans*, *K. lactis*, and *E. gossypii*; compared to 9–22 multigene families specific to each species (total 84). Interestingly, if ∼60% (51) of the latter correspond to genes present in single copies in the four other yeast species, suggesting lineage-specific duplications of ancestral genes, the rest (33) are absent from the four other yeast species, suggesting lineage-specific acquisitions. Tandem gene arrays show faster kinetics of evolutionary change consistent with their adaptive role (Brown *et al.* 1998; Müller *et al.* 2009). In the same five species above, only eight arrays were found entirely conserved, compared to 9–16 arrays specific to each species (total 63) and 32 others showing partial conservation.

The *de novo* formation of genes has traditionally been regarded as a nearly impossible mechanism because of the extremely low probability of obtaining a genetically meaningful sequence by single nucleotide changes from a random sequence. But ideas along this line are rapidly progressing for two reasons. First, there is no such thing in nature as a random DNA sequence, in yeasts as well as any other genome. Noncoding parts of genomes are not random nucleotide sequences but sequences inherited from previous generations that, in eukaryotes at least, are almost entirely transcribed. Using the ribosome profiling method, it was recently shown that the genome of *S. cerevisiae* contains a large number of *protogenes*, *i.e.*, short sequences susceptible to create new active genes after a limited number of point mutations, the logical symmetry to pseudogenes (Carvunis *et al.* 2012). Second, actual examples of *de novo* gene formation have now been experimentally demonstrated in *S. cerevisiae*: the *BSC4* gene encoding a DNA-repair enzyme active in stationary phase, and the *MDF1* gene encoding a pleiotropic protein regulating glucose assimilation and the budding pathway. The first gene has emerged in *S. cerevisiae* from the conserved transcribed intergene between *LYP1* and *ALP1*, two genes encoding amino-acid transporters themselves originated from an inverted tandem duplication of an ancestral gene followed by subfunctionalization (Cai *et al.* 2008). *BSC4* has 132 codons. The second gene was created in *S. cerevisiae* on the opposite strand of *ADF1*, a gene considered as its antisense regulator before it was discovered to be, in reality, the evolutionarily conserved ancestral gene present in all other species (Li *et al.* 2010, 2014). *MDF1* has 153 codons. The presence of a few *de novo* created genes in each yeast lineage is also expected from careful comparison between the *Lachancea* genomes (Vakirlis *et al.* 2016).

Finally, the capture of nonchromosomal elements can be regarded as an intrinsic mechanism of genome evolution when permanent elements are involved, or as involving horizontal exchanges (below) in other cases. The most conspicuous examples of the first case are represented by the copies of mtDNA fragments (NUMT) found in chromosomes of almost all yeast species studied so far (Sacerdot *et al.* 2008; Leh-Louis *et al.* 2012). Depending upon their nature and precise location, such elements may alter genes in a variety of manners, including the possibility to extend ancestral reading frames. Their insertion occurs during repair of chromosomal double-strand breaks, as experimentally demonstrated in *S. cerevisiae* several years ago (Ricchetti *et al.* 1999).

#### Horizontal exchanges and interspecies hybridization

Similarly, traces of DNA plasmids or RNA viruses (designated NUPAV) were recognized in 40% of the 20 Saccharomycotina genomes examined (Frank and Wolfe 2009), probably representing ancient captures. Genomes of several species of the CTG clade contain a capsid protein gene originating from double-stranded RNA viruses (Taylor and Bruenn 2009). The evolutionary role of such phenomena in yeasts is not yet fully understood but, as mentioned above, such mechanisms were probably at the origin of point centromeres and mating-type switching mechanisms in the Saccharomycetaceae.

In addition to the above cases, yeast genomes show numerous traces of other horizontal inheritance without which their evolution would not have been the same. First, a number of genes, or sometimes gene clusters, have been acquired from other organisms. Second, a rapidly growing number of interspecies hybrids have now been recognized in various yeast lineages, raising the question of the degree of reticulation in yeast phylogenies.

Genes of bacterial origin, as judged from molecular phylogeny, have been recognized in almost every yeast genome sequenced so far, most of which correspond to basic metabolic functions. Their function in yeasts is not always demonstrated, but spectacular examples can be mentioned such as the acquisition of the *URA1* gene in the Saccharomycetaceae family (Gojković *et al.* 2004) or of β-lactamases in *Kuraishia capsulata* (Morales *et al.* 2013) and *B. adeninovorans* (Kunze *et al.* 2014). The *URA1* gene, encoding a dihydro-orotate dehydrogenase, has been critical in the evolution of the Saccharomycetaceae by allowing their propagation under strictly anaerobic conditions; the enzyme encoded by the ancestral gene *URA9* requiring oxygen for the synthesis of uracil. Similarly, the acquisition of genes encoding amino-acid racemases was also probably significant in the evolution of the lineages where it occurred (Fitzpatrick *et al.* 2008). The fact that horizontally acquired genes have a tendency to duplicate in several copies in the host genomes (Rolland *et al.* 2009) also supports the idea of functional selection.

Next to single genes, whose small size distribution suggests that they were acquired by natural DNA transformation (Rolland *et al.* 2009), yeast genomes also bear long chromosomal segments of alien origin. As already mentioned in *What Did We Learn from Comparative and Population Genomics of the Saccharomyces Species Complex?*, some wine strains of *S. cerevisiae* were found to contain a 17-kb-long segment containing 5 genes from *Zygosaccharomyces bailii* (Galeote *et al.* 2011) and a 65-kb-long segment containing 18 genes from *Torulaspora microellipsoides* (Marsit *et al.* 2015), two species from the ZT clade of Saccharomycetaceae present as contaminants in wine fermentations. The near complete nucleotide sequence identity of these segments (>99.7%) with respective donors demonstrate recent transfers into *S. cerevisiae* by a mechanism that remains to be identified. The circular permutations observed for the 17-kb segment of *Z. bailii* suggests that, in this case, an extrachromosomal circular element served as a vector between the two yeast species. Another remarkable example of introgression of long chromosomal segments of alien origin is represented by the presence of nitrate-assimilating gene clusters in several members of the methylotroph subgroup of Saccharomycotina (Morales *et al.* 2013). As a result, these yeasts assimilate nitrate, an important trait acquired by an ancestral introgression from plants or other nitrate-assimilating fungi. Again, the mechanism at the origin of such a large introgression remains to be elucidated.

Finally, it is now clear that interspecies hybrids, for a long time considered as evolutionary dead ends because of their low meiotic fertility, play a more important role than anticipated in yeast genome evolution (Morales and Dujon 2012). The existence of hybrids in *Saccharomyces* yeasts was suspected long ago for major brewing strains (see *What Did We Learn from Comparative and Population Genomics of the Saccharomyces Species Complex?*). It has now been fully demonstrated by genome sequencing (Nakao *et al.* 2009; Wahlter *et al.* 2014). Spontaneous interspecies hybridization appears so frequent in this genus, at least under industrial conditions of fermentation, that a complex hybrid was for long time mistaken for the type strain of *S. bayanus* before actual parental species were discovered (Libkind *et al.* 2011; Nguyen *et al.* 2011). This raises the important question of how to recognize hybrid yeast genomes after they have been resolved in mosaics of parental pieces (see below) in the absence of information on the parents. It is quite possible, therefore, that the evolution of yeast genomes includes a higher degree of reticulation than generally expected. This is particularly true because meiotically fertile lines can rapidly emerge from initially unfertile hybrids, using different mechanisms (Greig *et al.* 2002a,b; Sebastiani *et al.* 2002; Antunovics *et al.* 2005).

The formation of interspecies hybrids appears as a general phenomenon in all subgroups of Saccharomycotina. Natural hybrid genomes were found in *Z. rouxii* (Gordon and Wolfe 2008) and *Z. bailii* (Mira *et al.* 2014), two members of the ZT clade of Saccharomycetaceae; in *Saccharomycopsis fibuligera* (Choo *et al.* 2016); in members of the CTG clade such as *Millerozyma sorbitophila* (Leh-Louis *et al.* 2012) and *Candida orthopsilosis* (Pryszcz *et al.* 2014; Schröder *et al.* 2016); as well as in *Dekkera bruxellensis* (Curtin *et al.* 2012; Borneman *et al.* 2014), a representative of the methylotroph subgroup. The phenomenon also extends to Basidiomycota yeasts where virulent hybrids were found in *Cryptococcus* (Boekhout *et al.* 2001; Cogliati *et al.* 2006; Bovers *et al.* 2008; Li *et al.* 2012).

As for the *Saccharomyces*, hybridizations involved parents of distinct ploidy, creating diploid or triploid hybrid genomes that may subsequently evolve into a variety of aneuploidies by partial chromosome loss. In general, the evolution of hybrid strains during their clonal development remains to be better studied. In this regard, the osmotolerant yeast *M. sorbitophila* proved informative because this genome, whose parents differed from each other by 12–15% nucleotide sequence divergence, is in the process of resolution by a mechanism of loss of heterozygosity that transformed some segments of chromosomes or even entire chromosomes in homozygous pairs (Leh-Louis *et al.* 2012). The loss-of-heterozygosity mechanism must also operate in other hybrids and even in wild diploid *S. cerevisiae* strains if one judges from the frequent presence of long homozygous segments in pairs of chromosomes (Belloch *et al.* 2009; Nakao *et al.* 2009; Magwene *et al.* 2011). The fact that these segments almost always extend to chromosome ends suggests that they result from a break-induced mechanism during mitotic divisions (Morales and Dujon 2012) or from reversed meiotic prophases (Laureau *et al.* 2016). Interestingly, in *M. sorbitophila* as well as in other hybrids carefully studied, only one of the two parental rDNA loci persists, suggesting a rapid resolution in favor of one parent immediately after hybridization. This is interesting to keep in mind because the sequences of the rDNA are being used to define yeast species (Kurtzman *et al.* 2011)and therefore a number of hybrid genomes may have remained undetected so far. The 1-Mb-long, GC-rich segment in the genome of all *L. kluyveri* isolates sequenced so far (Friedrich *et al.* 2015) suggests such a “species” with several ancestries. Subsequent studies will be necessary to precisely determine how reticulated yeast genome evolution was.

## Perspectives

Much more has been learned in a short time from the remarkable progress of yeast genomics than what could be anticipated at the time of the original sequencing of the *S. cerevisiae* genome, with lessons largely applicable to all eukaryotic organisms. Reasons for this success lie of course in the facility to sequence many yeast genomes using modern technologies but also because yeasts offer a rare combination of exquisite experimental systems and widespread natural populations spanning a broad range of evolutionary distances, ecological diversity, and interactions with human activities. It can easily be anticipated that this short review will be quickly outdated by a rapidly expanding set of novel data. But the basic questions elucidated or unraveled by yeast genomics offer a solid background for the future of genome biology. What is the relative importance of sequence variation, chromosomal rearrangements, copy number variation, and gene gain and loss in speciation and in the phenotypic variation within populations? What is the importance of horizontal exchanges, therefore of extant biodiversity and ecological proximity, in these phenomena and in the origin of successful lineages? And what is the impact of human activities on these questions? At the same time, yeast genomes continue to spring surprises. The genetic code is more variable than expected. Mobile elements find their way across distinct lineages. Novel genes are generated *de novo*. And all this in a group of organisms, the *Saccharomycotina*, whose long-term success seems to have been based on the ancestral loss of essential functions relative to RNA processing or chromatin modification that appear so critical to other eukaryotes. It can only be hoped that more research in this field will continue for a long time.
